# Genome-wide association analysis unveils novel QTLs for seminal root system architecture traits in Ethiopian durum wheat

**DOI:** 10.1186/s12864-020-07320-4

**Published:** 2021-01-06

**Authors:** Admas Alemu, Tileye Feyissa, Marco Maccaferri, Giuseppe Sciara, Roberto Tuberosa, Karim Ammar, Ayele Badebo, Maricelis Acevedo, Tesfaye Letta, Bekele Abeyo

**Affiliations:** 1 Department of Microbial, Cellular and Molecular Biology, Addis Ababa University, P.O.Box 1176, Addis Ababa, Ethiopia; 2 Department of Biology, Debre Tabor University, Debra Tabor, Ethiopia; 3 Department of Agricultural and Food Sciences, University of Bologna, Bologna, Italy; 4 International Maize and Wheat Improvement Center (CIMMYT), Texcoco, Mexico; 5 International Maize and Wheat Improvement Center (CIMMYT), Addis Ababa, Ethiopia; 6 International Programs, College of Agriculture and Life Sciences, Cornell University, New York City, NY USA; 7 Oromia Agricultural Research Institute, Addis Ababa, Ethiopia

**Keywords:** Ethiopian durum wheat, Root system architecture, QTL, GWAS, SNP

## Abstract

**Background:**

Genetic improvement of root system architecture is essential to improve water and nutrient use efficiency of crops or to boost their productivity under stress or non-optimal soil conditions. One hundred ninety-two Ethiopian durum wheat accessions comprising 167 historical landraces and 25 modern cultivars were assembled for GWAS analysis to identify QTLs for root system architecture (RSA) traits and genotyped with a high-density 90 K wheat SNP array by Illumina.

**Results:**

Using a non-roll, paper-based root phenotyping platform, a total of 2880 seedlings and 14,947 seminal roots were measured at the three-leaf stage to collect data for total root length (TRL), total root number (TRN), root growth angle (RGA), average root length (ARL), bulk root dry weight (RDW), individual root dry weight (IRW), bulk shoot dry weight (SDW), presence of six seminal roots per seedling (RT6) and root shoot ratio (RSR). Analysis of variance revealed highly significant differences between accessions for all RSA traits. Four major (− log_10_*P* ≥ 4) and 34 nominal (− log_10_*P* ≥ 3) QTLs were identified and grouped in 16 RSA QTL clusters across chromosomes. A higher number of significant RSA QTL were identified on chromosome 4B particularly for root vigor traits (root length, number and/or weight).

**Conclusions:**

After projecting the identified QTLs on to a high-density tetraploid consensus map along with previously reported RSA QTL in both durum and bread wheat, fourteen nominal QTLs were found to be novel and could potentially be used to tailor RSA in elite lines. The major RGA QTLs on chromosome 6AL detected in the current study and reported in previous studies is a good candidate for cloning the causative underlining sequence and identifying the beneficial haplotypes able to positively affect yield under water- or nutrient-limited conditions.

**Supplementary Information:**

The online version contains supplementary material available at 10.1186/s12864-020-07320-4.

## Background

Ethiopian farmers have grown tetraploid wheat (*Triticum turgidum ssp. durum*) since its introduction in the northern highlands of the country around 3000 BC [1]. Cultivation was mostly under adverse environmental conditions that likely favored the development of a broad gene pool of durum wheat landraces adapted to various environmental conditions. Ethiopian durum wheat landraces provide a rich and yet untapped native biodiversity [2]. Vavilov [3] and Zohary [4] reported the presence of high-genetic diversity in cultivated tetraploid wheat and recent studies highlighted the uniqueness of Ethiopian durum landraces from the Fertile Crescent collections (primary center of domestication) and considered Ethiopia as a possible second domestication center for the crop [5]. Previous studies, carried out with phenotypic [2, 6–8] and molecular approaches [9–12], have indicated Ethiopian durum germplasm to be a highly diverse and potentially unique source of valuable traits [13–15]. This is basically due to the wide range of agro-ecological conditions (altitude in a range of 1600 to 3000 masl) coupled with diverse farmers’ culture [9]. Notably, more than 7000 Ethiopian durum wheat landrace accessions are conserved in the Ethiopian Biodiversity Institute (EBI) gene bank [16]. However in recent time, durum wheat cultivation has been largely replaced by bread wheat varieties developed from international and national breeding programs throughout the country [17].

Roots play a key role in nutrient and water uptake, soil anchoring and mechanical support, storage functions, and as the major interface between the plant and various biotic and abiotic factors in the soil environment. Root system architecture (RSA) describes the shape and structure of the root system, both of which have great functional importance [18, 19] and plays a pivotal role in crop performance, especially for cultivation under non-optimal nutritional and water source conditions [20–22]. Due to recurrent climate change, declining of soil fertility and water availability, enhancing the genetic capacity to capture the available soil resources is considered a primary target for breeding resource-use efficient crops [20, 23, 24]. Hence, RSA has been an active research topic for the last couple of decades and since then different RSA ideotypes have been proposed and investigated in crops [25–27]. The narrow-and-deep or wide-and-shallow root ideotypes have been studied for their effects in nutrient acquisition and drought resistance in crops [28–31]. Deep and narrow-angled roots could allow plants to exploit more effectively water and nitrogen that are often found in deeper soil layers [29, 30, 32], while shallow wider angled roots enable plants to more effectively uptake nutrients such as phosphorous that are abundantly found at shallower depths in the soil [33].

The genetic basis of RSA traits in durum wheat has been investigated with both linkage and association mapping using durum wheat recombinant inbred line (RIL) populations and/or elite durum wheat panels suitable for association mapping [19, 21, 34–37]. This notwithstanding, beside the recent studies by Roselló et al. [38] and Ruiz et al. [39], durum wheat landraces have not been extensively studied so far. Ethiopian durum wheat landraces are particularly rich in genetic diversity and thus are very valuable to dissect the genetic basis of governing the variability of RSA traits. Hence, this study aimed to conduct a genome-wide association analysis for root system architecture traits in Ethiopian durum wheat comprising historical landraces (167) and modern cultivars (25) to identify RSA quantitative trait loci (QTLs) of potential interest for marker-assisted selection.

## Results

### Phenotypic variation among RSA traits

A total of 2880 seedlings and 14,947 seminal roots were processed and measured for various RSA traits (Additional file 2: Table S2). Analysis of variance (ANOVA) for the studied RSA traits is presented in Table 1**.**

**Table 1:** ANOVA and heritability results for the root system architecture traits measured in 12-day-old seedlings of 192 Ethiopian durum wheat accessions

Traits	TRL (cm)	ARL (cm)	RGA (°)	TRN (n)	RDW (mg)	IRW (mg)	SDW (mg)	RSR (ratio)	RT6 (%)
**Mean**	135.3	26.1	97.3	5.1	60.5	11.6	69.5	0.87	37.2
**Max**	195.4	36.9	130.5	6.7	115.0	21.4	116.6	1.32	100
**Min**	66.2	16.5	45.7	3.4	27.7	5.9	34.7	0.67	0
***h***^**2**^ **(%)**	88.97	91.0	74.3	75.1	91.3	89.9	90.4	71.3	67.0
**CV (%)**	11.1	8.4	14.6	8.1	14.2	14.6	11.5	10.5	__^a^
***P*** **accessions**^**b**^	***	***	***	***	***	***	***	***	***
**Replicates**^**c**^	NS	NS	NS	*	*	*	NS	*	*

^**a**^ Not reported due to the presence of many values equal to 0.00

^**b**^ Significance of the difference between accessions

^**c**^ Significance of the difference between replicates

*NS* non-significant

* *P* < 0.05; *** *P* < 0.001

See Table 6 for trait abbreviations

**Fig. 1:**
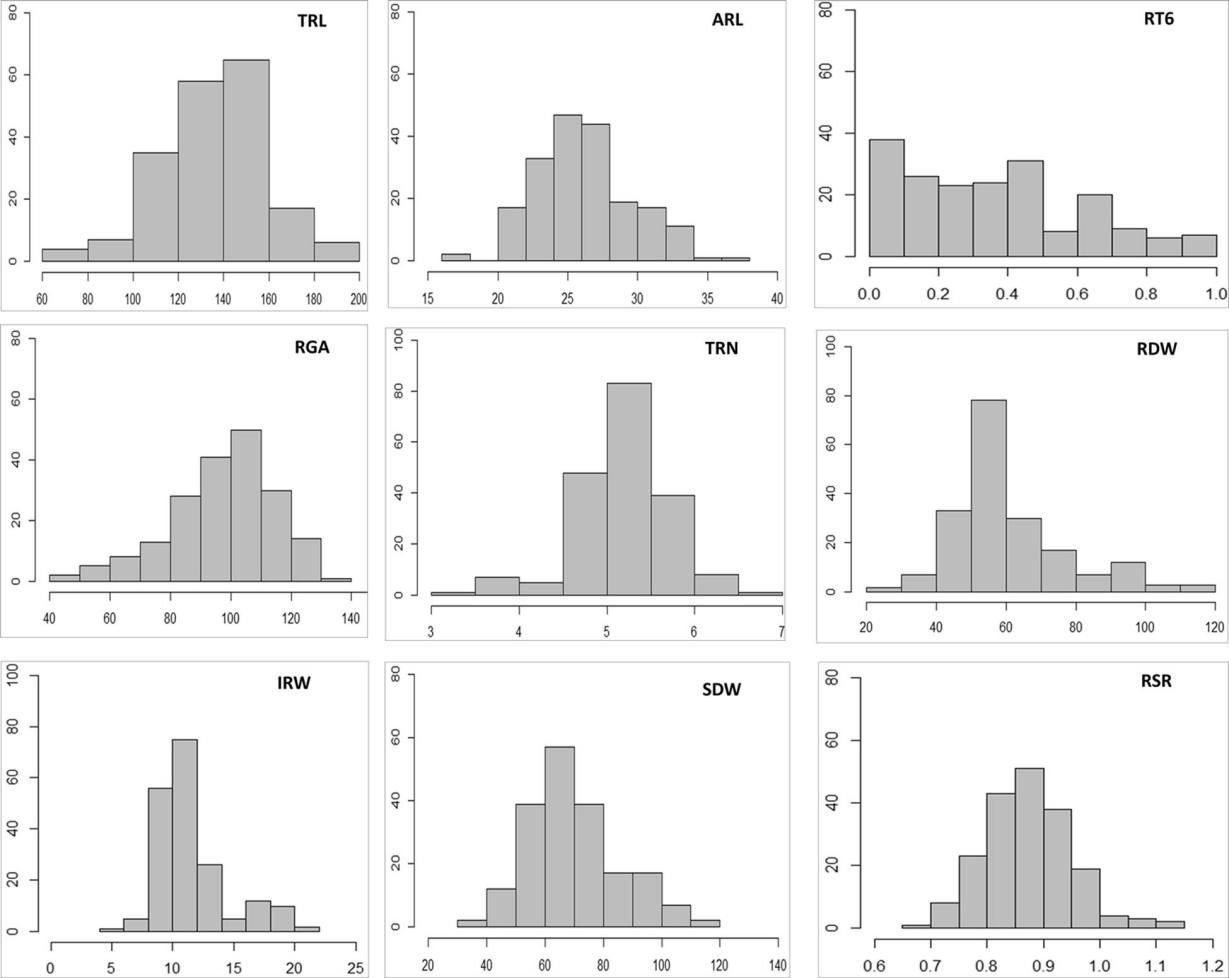
Distribution frequency for RSA traits measured from 12-day-old seedlings in 192 Ethiopian durum wheat accessions. See Table 6 for trait abbreviations

The ANOVA results indicate the presence of highly significant variation among accessions for all RSA traits. In particular, the seminal root angle ranged from 45.7 to 130.5° with a mean value of 97.3° while the total and average root length and number of roots ranged from minimum values of 66.2 cm, 16.5 cm and 3.4 to maximum values of 195.4 cm, 36.9 cm and 6.7, respectively. The root and shoot dry weight varied from minimum values of 27.7 and 34.7 g to maximum values of 115.0 and 116.6 g, respectively. The coefficient of variance (CV) of RSA traits ranged from 8.38% for average root length (ARL) to 14.63 for root growth angle (RGA). Individual root dry weight (IRW) and bulk root dry weight (RDW) also scored high CV, with a value of 14.55 and 14.22%, respectively. The frequency distribution of most RSA traits was normal except for RT6 that showed a bi-modal distribution **(**Fig. 1**)**.

Most RSA traits showed high level of broad sense heritability (*H*^2^). Bulk root dry weight (RDW), average root length (ARL) and bulk shoot dry weight (SDW) showed the top three values (91.3, 91.0 and 90.4%, respectively) while the presence of the 6th root showed the lowest value (67.0%).

### Correlation among RSA traits

**Fig. 2:**
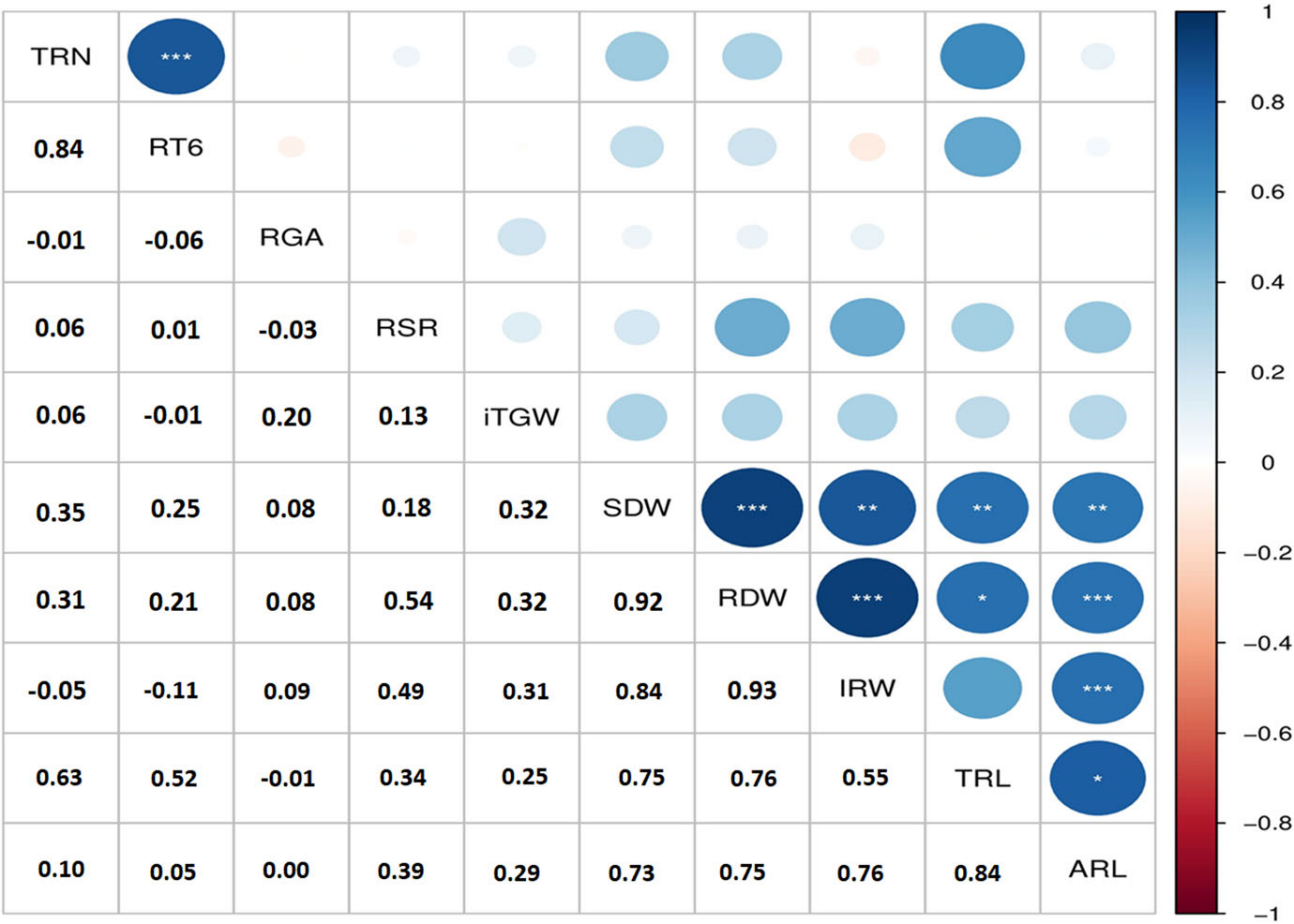
Correlation coefficient and level of significant for the initial thousand grain weight and RSA traits measured in 12-day-old seedlings of 192 Ethiopian durum wheat accessions

Several strong correlations were observed between RSA traits (Fig. 2). Highly significant positive correlations were detected for RDW vs. IRW (0.93), RDW vs. SDW (0.92) and IRW vs. SDW (0.84). Strong correlations were recorded between TRN and RT6; TRL and ARL with a correlation coefficient of 0.84 and 0.82, respectively. The initial thousand grain weight showed no significant correlation with any RSA trait suggesting that variation of RSA traits did not have maternal etiology caused by variation in seed size.

Landraces showed a wider range of variability than cultivars in most RSA traits although the latter outperformed the former for some traits (Table 2 and Additional file 9: Figure S2.). For instance, the cultivars mean values for root and shoot dry weight were 90.3 and 92.8 mg, while landraces scored only 56.9 and 66.5 mg for the same traits, respectively. Cultivars also performed better than landraces for TRL and ARL while TRN and RT6 were the only two RSA traits for which landraces showed slightly higher mean values than cultivars.

**Table 2:** Mean and range values of 25 cultivars and 167 landraces for RSA traits

Accession type	Sample size		iTGW (mg)	TRL (cm)	ARL (cm)	RGA (°)	TRN (n)	RDW (mg)	IRW (mg)	SDW (mg)	RSR (ratio)	RT6 (%)
**Cultivar**	25	Mean	45.90	161.50	31.84	99.79	5.09	90.29	17.61	92.81	0.97	0.30
		Max	52.52	195.46	36.90	124.17	5.66	115.02	21.48	116.69	112.82	0.77
		Min	33.92	103.29	26.02	72.20	3.73	52.02	11.31	65.22	76.54	0
**Landrace**	167	Mean	42.28	132.01	25.40	98.06	5.20	56.89	10.80	66.49	0.86	0.38
		Max	61.67	194.40	32.60	130.53	6.75	100.55	19.21	102.29	104.63	1
		Min	29.55	66.26	16.58	45.76	3.47	27.71	5.94	34.76	67.98	0

See Table 6 for trait abbreviations

### Population structure and linkage disequilibrium decay analysis

**Fig. 3:**
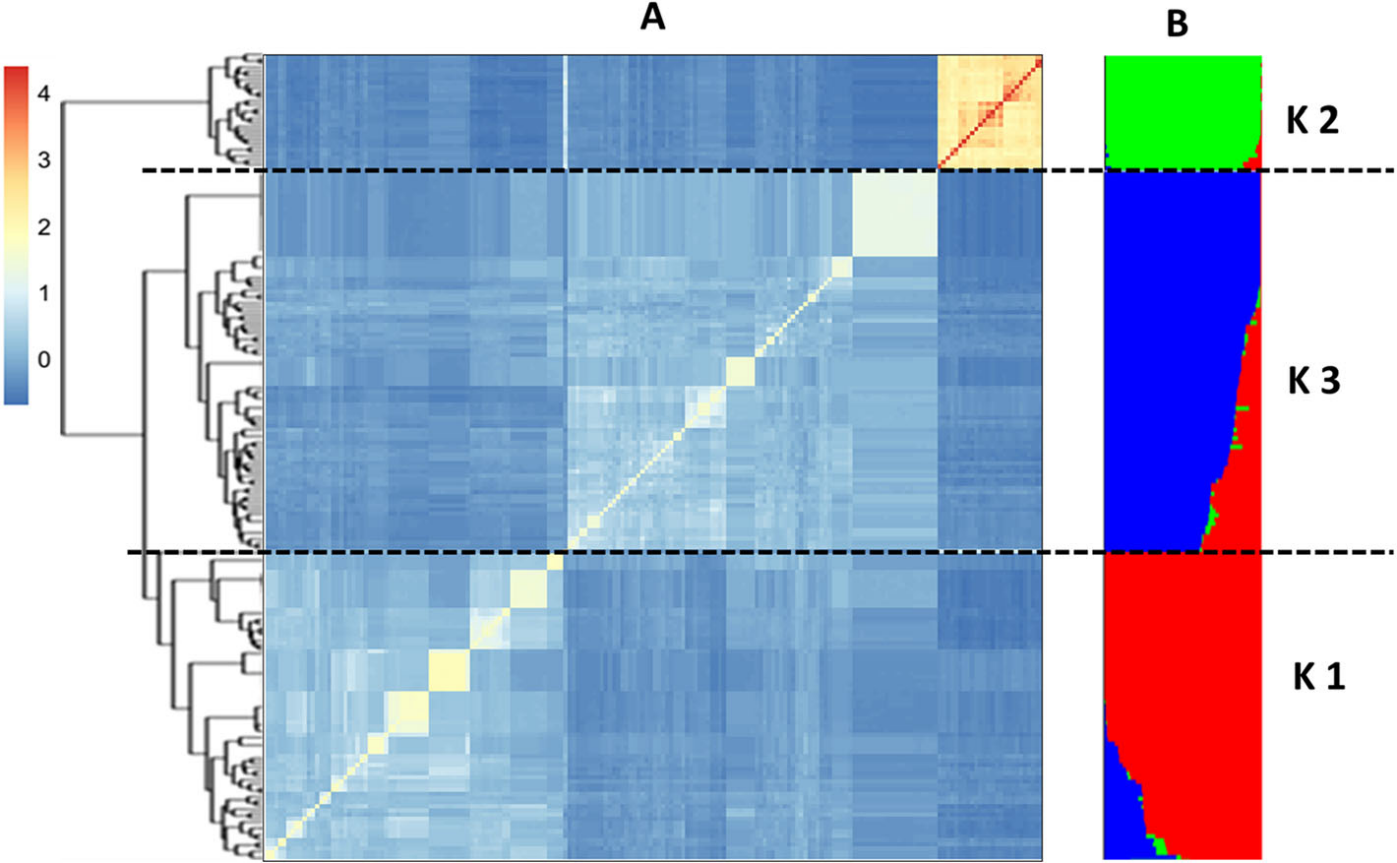
Population structure and kinship-matrix similarity analysis for 192 Ethiopian durum wheat accessions. Heat-map clustering results based on the kinship matrix from tag-SNP (r^2^ = 1) by identity-by-state (IBS) algorithm (**a**). Population structure plot and K1, K2 and K3 represents subpopulations 1, 2 and 3, respectively (**b**). The black-dash lines separated the panel into three subpopulations. Accessions arrangement was based on the order of heat-map kinship result. The color represents the membership of each accession in the STRUCTURE-inferred subpopulations. The color of the legend indicates the level of kinship similarity of the heat-map

According to population structure analysis, the panel was subdivided into three subpopulations of 75, 27 and 90 accessions each (Fig. 3a, b and Additional file 3: Table S3). All 26 cultivars clustered into subpopulation 2 except for ‘Selam’ that grouped in subpopulation 1. Clustering analysis indicated that SNP data failed to group landraces clearly based on their geographical backgrounds and accessions were admixed into the three subpopulations irrespective of their geographic origin. Box plot of the three sub-populations inferred from STRUCTURE analysis for the mean values of RSA traits is reported in Additional file 9: Figure S3.

The mean genome wide *r*^2^ value was 0.12, with 55% of the pair-wise linkage disequilibrium comparisons showing significant association at *P* < 0.01. Chromosome 3B scored the highest mean value (*r*^2^ = 0.19) with 64% significant pair-wise LD comparisons. On the other hand, 7A scored the lowest mean *r*^2^ value (0.11) and 48% of pairwise LD comparisons were significant. The genome-wide LD decayed below *r*^2^ = 0.3 (the standard critical threshold) at 2.25 cM. This defines the ±2.25 cM as the genome-wide critical distance to detect linkage and, therefore, as the QTL confidence interval around the QTL-tag SNP, i.e. the SNP found at the peak of the corresponding QTL. The specific critical *r*^2^ value beyond which LD is due to true physical linkage was 0.15 and the intersect of the threshold with the LD decay curve was at 5.75 cM.

### GWAS analysis of RSA traits

After filtering SNP data and following imputation, a total of 10,789 polymorphic SNP markers (4591 and 6198 SNPs from A and B genomes, respectively) were used for marker-traits association (MTA) analyses. The mixed linear model with population structure and kinship matrix was chosen for MTA analysis, as the quantile-quantile (Q-Q) plot showed that the observed MTA *P*-values were close to the expected distribution (Additional file 9: Figure S4). A total of 275 QTLs with various significant values were identified for the tested RSA traits. The only four major QTLs above the experiment-wise threshold (− log_10_*P* ≥ 4) were *EPdwRGA-6A*, *EPdwRDW-4A*, *EPdwiTGW-3B.1* and *EPdwIRW-5A* with values of 6.85, 4.34, 4.15 and 4.06 which accounted for 16.08, 8.41, 8.71 and 8.03% of the phenotypic variation, respectively. Thirty-four QTLs reached the marker-wise threshold of – log_10_*P* ≥ 3 in which the highest number was identified for TRN with eight QTLs followed by SDW and IRW each with six nominal QTLs. Additionally, three nominal QTLs were identified for TRL, iTGW and RT6, two for RDW and only one for RGA, ARL and RSR. The other 237 QTLs with a marker-wise threshold of – log_10_*P* ≥ 2 were identified as suggestive QTLs. The major and nominal QTLs are reported in Table 3 while the complete list of identified QTLs with the marker-wise threshold value of –log_10_*P* ≥ 2 are reported in Additional file 4: Table S4. Thirteen markers showed significant associations for more than one RSA trait that could be due to either a pleiotropic effect or tight linkage, hence considered as separate QTLs for corresponding traits (Table 4**)**. Notably, the root growth angle QTL showed limited overlap with QTLs of other RSA traits.

**Table 3:** List of major and nominal QTLs for RSA traits identified in 192 Ethiopian durum wheat accessions

QTL	Trait^**a**^	Marker^**b**^	Chr	Position (cM)	*P*-value	– log_10_*P*	R^**2**^ (%)	Sig. SNPs^**c**^	CI (cM)^**d**^	Allele (SNP base)	Effect	Allele count (No.)	Allele (SNP base)	Effect	Allele count (No.)
*EPdwTRL-1B*	TRL	*IWB60732*	1B	33.75	0.000349	3.46	6.75	0	31.5–36	A	16.08	41	G	0	151
*EPdwTRL-4B*^e^	TRL	*IWB23476*	4B	114.43	0.000761	3.12	5.67	0	112.18–116.68	A	8.8	154	G	0	38
*EPdwTRL-5A*	TRL	*IWA3196*	5A	16.83	0.000845	3.07	5.57	1	14.58–19.08	C	39.84	126	T	0	66
*EPdwARL-2A*^e^	ARL	*IWB53380*	2A	35.63	0.001	3	5.72	3	33.38–37.88	A	4.76	159	G	0	33
*EPdwRGA-6A*	RGA	*IWB71119*	6A	122.43	1.42E-07	6.85	16.08	8	120.18–124.68	C	−22.31	113	T	0	79
*EPdwRGA-4A*^e^	RGA	*IWB69385*	4A	167.15	0.000703	3.15	6.38	1	164.90–169.94	G	13.44	78	T	0	114
*EPdwTRN-1A.1*	TRN	*IWB8696*	1A	5.2	0.00085	3.07	5.71	0	2.95–7.45	A	0.78	31	G	0	161
*EPdwTRN-1A.2*	TRN	*IWB12589*	1A	13.62	0.00046	3.34	6.32	2	11.37–15.87	A	0.57	16	G	0	176
*EPdwTRN-1B*	TRN	*IWB35568*	1B	27.21	0.00041	3.39	6.43	1	24.96–29.46	A	0.54	111	G	0	81
*EPdwTRN-4A*	TRN	*IWB21309*	4A	17.01	0.000204	3.69	7.13	3	14.76–19.26	C	−0.43	87	T	0	105
*EPdwTRN-4B.1*	TRN	*IWB10265*	4B	44.92	0.000724	3.14	5.87	1	42.67–47.17	A	0.39	138	G	0	54
*EPdwTRN-4B.2*	TRN	*IWB35047*	4B	80.4	0.000256	3.59	6.90	0	78.15–82.65	C	−0.44	91	G	0	101
*EPdwTRN-4B.3*	TRN	*IWB66095*	4B	91.74	0.000429	3.37	6.39	4	89.49–93.99	C	0.52	47	T	0	145
*EPdwTRN-7A*	TRN	*IWB3767*	7A	146.9	0.000844	3.07	5.72	3	144.65–149.15	C	0.5	171	T	0	21
*EPdwRDW-1B*	RDW	*IWB60732*	1B	33.75	0.00028	3.55	6.72	1	31.50–36.00	A	10.59	41	G	0	151
*EPdwRDW-3A*^e^	RDW	*IWB67049*	3A	80.8	0.000682	3.17	5.76	54	78.55–83.05	A	−14.1	15	G	0	177
*EPdwRDW-4A*^e^	RDW	*IWB21309*	4A	17.01	4.59E-05	4.34	8.41	3	14.76–19.26	C	−9.81	87	T	0	105
*EPdwSDW-1A*	SDW	*IWB29244*	1A	123.21	0.000465	3.33	6.27	2	120.96–125.46	C	−9.18	138	T	0	54
*EPdwSDW-1B*	SDW	*IWB60732*	1B	33.75	0.000419	3.38	6.58	0	31.50–36.00	A	13.4	41	G	0	151
*EPdwSDW-3B*^e^	SDW	*IWB35437*	3B	41.34	0.000796	3.10	5.93	2	39.09–43.59	C	−17.57	36	T	0	156
*EPdwSDW-4A*^e^	SDW	*IWB21309*	4A	17.01	0.000151	3.82	7.39	3	14.76–19.26	C	−8.2	87	T	0	105
*EPdwSDW-4B*	SDW	*IWB23476*	4B	114.43	0.000594	3.23	6.03	1	112.18–116.68	A	15.41	154	C	0	38
*EPdwSDW-5B*	SDW	*IWB8808*	5B	190.51	0.000809	3.09	5.73	0	188.26–192.76	A	−9.41	36	C	0	156
*EPdwiTGW-3B.1*	iTGW	*IWB65507*	3B	126.23	7.09E-05	4.15	8.71	2	123.98–128.48	A	−9.84	160	G	0	32
*EPdwiTGW-3B.2*	iTGW	*IWB11298*	3B	129.61	0.000135	3.87	8.01	12	127.36–131.86	A	10.94	160	C	0	32
*EPdwiTGW-7A*	iTGW	*IWB7752*	7A	98.3	0.000448	3.35	6.73	1	96.05–100.55	A	−7.27	173	G	0	19
*EPdwiTGW-7B*	iTGW	*IWB60*	7B	33.71	0.000486	3.31	6.64	1	31.46–35.96	A	3.83	28	C	0	164
*EPdwIRW-1B*	IRW	*IWB60732*	1B	33.75	0.000789	3.10	5.80	0	31.50–36.0	A	1.70	41	G	0	151
*EPdwIRW-2B*	IRW	*IWB29332*	2B	160.51	0.000328	3.48	6.68	0	158.26–162.76	A	4.50	24	G	0	168
*EPdwIRW-5A*	IRW	*IWA3196*	5A	16.83	8.64E-05	4.06	8.03	0	14.58–19.08	C	7.54	26	T	0	166
*EPdwIRW-5B.1*^e^	IRW	*IWB70122*	5B	40.39	0.000702	3.15	5.92	7	38.14–12.64	A	2.81	16	C	0	176
*EPdwIRW-5B.2*^e^	IRW	*IWA332*	5B	16.73	0.00101	3	5.56	1	14.48–18.89	G	−2.96	15	T	0	177
*EPdwIRW-6B*^e^	IRW	*IWB73456*	6B	90.33	0.00046	3.34	6.34	4	88.08–92.58	C	2.94	21	T	0	171
*EPdwIRW-7A*^e^	IRW	*IWB11841*	7A	94.72	0.000684	3.17	5.94	0	92.47–96.97	C	−1.43	66	T	0	126
*EPdwRSR-3A*	RSR	*IWB25948*	3A	96.93	0.000947	3.02	4.62	2	94.68–99.18	C	−0.10	170	T	0	22
*EPdwRT6-4B.1*^e^	RT6	*IWB72884*	4B	45.01	0.000211	3.68	7.13	7	42.76–47.26	A	0.21	138	G	0	54
*EPdwRT6-4B.2*^e^	RT6	*IWB35047*	4B	80.4	0.000351	3.45	6.62	0	78.15–82.65	C	−0.22	91	G	0	101
*EPdwRT6-4B.3*^e^	RT6	*IWB66095*	4B	91.74	0.000481	3.32	6.30	3	89.49–93.99	C	0.28	47	T	0	145

^**a**^ RSA trait acronyms: iTGW, initial thousand grain weight; TRL, total root length; ARL, average root length; RGA, root growth angle; TRN, total root number; RDW, root dry weight; IRW, individual root dry weight; SDW, shoot dry weight; RSR, root to shoot ratio; RT6, presence of the sixth root

^**b**^ SNP found at the peak of corresponding QTL (QTL-tag SNP)

^**c**^ The number of significant SNPs present in the significant interval

^**d**^ Confidence interval flanking the QTL-tag SNP based on the tetraploid wheat consensus map of Maccaferri et al. (2015)

^e^ The fourteen newly discovered RSA QTLs

See Table 6 for trait abbreviations

**Table 4:** Markers with a significant association/concurrent effect on more than one RSA trait

Marker^a^	QTL	Chr	Position (cM)	Trait^b^	– log_**10**_***P***	R^**2**^ (%)	CI (cM)
*IWB29244*	*EPdwSDW-1A*	1A	123.21	SDW	3.3	6.3	120.96–125.46
				RDW	2.3	3.9	
*IWB60732*	*EPdwRDW-1B*	1B	33.75	RDW	3.6	6.7	31.5–36
	*EPdwSDW-1B*			SDW	3.4	6.6	
	*EPdwTRL-1B*			TRL	3.5	6.8	
	*EPdwIRW-1B*			IRW	3.1	5.8	
*IWB35568*	*EPdwTRN-1B*	1B	27.21	TRN	3.4	6.4	24.96–29.46
				RT6	2.2	3.8	
*IWB53380*	*EPdwARL-2A*	2A	35.63	ARL	3.0	5.7	33.38–37.88
				IRW	2.0	3.4	
				RDW	2.4	4.1	
				SDW	2.6	4.9	
				TRL	2.8	5.2	
*IWB29332*	*EPdwIRW-2B*	2B	160.51	IRW	3.5	6.7	158.26–162.76
				TRL	2.1	2.12	
*IWB67049*	*EPdwRDW-3A*	3A	80.80	RDW	3.2	5.8	78.55–83.05
				SDW	2.4	4.3	
*IWB35437*	*EPdwSDW-3B*	3B	41.34	SDW	3.1	5.9	39.09–43.59
				RDW	2.6	4.9	
				IRW	2.2	3.7	
*IWB21309*	*EPdwRDW-4A*	4A	17.01	RDW	4.3	8.4	14.76–19.26
	*EPdwSDW-4A*			SDW	3.8	7.4	
	*EPdwTRN-4A*			TRN	3.7	7.1	
				RT6	2.7	4.9	
*IWB35047*	*EPdwTRN-4B.2*	4B	80.41	TRN	3.6	6.9	78.15–82.65
	*EPdwRT6-4B.2*			RT6	3.5	6.6	
				SDW	2.3	4.0	
				RDW	2.1	3.5	
*IWB66095*	*EPdwTRN-4B.3*	4B	91.74	TRN	3.4	6.4	89.49–93.99
	*EPdwRT6-4B.3*			RT6	3.3	6.3	
*IWB23476*	*EPdwSDW-4B*	4B	114.43	SDW	3.2	6.0	112.18–116.68
	*EPdwTRL-4B*			TRL	3.1	5.7	
				IRW	2.4	4.3	
				RDW	2.2	3.7	
*IWA3196*	*EPdwIRW-5A*	5A	16.83	IRW	4.1	8.0	14.58–19.08
	*EPdwTRL-5A*			TRL	3.1	5.6	
				RDW	2.1	3.4	
*IWB11841*	*EPdwIRW-7A*	7A	94.72	IRW	3.2	5.9	92.47–96.97
				TRL	2.9	5.4	
				RDW	2.4	4.1	

^a^ The SNP found at the peak of the corresponding QTL (QTL-tag SNP) for group of RSA traits

^b^ Cluster of RSA traits significantly associated with QTL-tag SNPs. See Table 6 for trait abbreviations

### QTL clusters for RSA traits

**Fig. 4:**
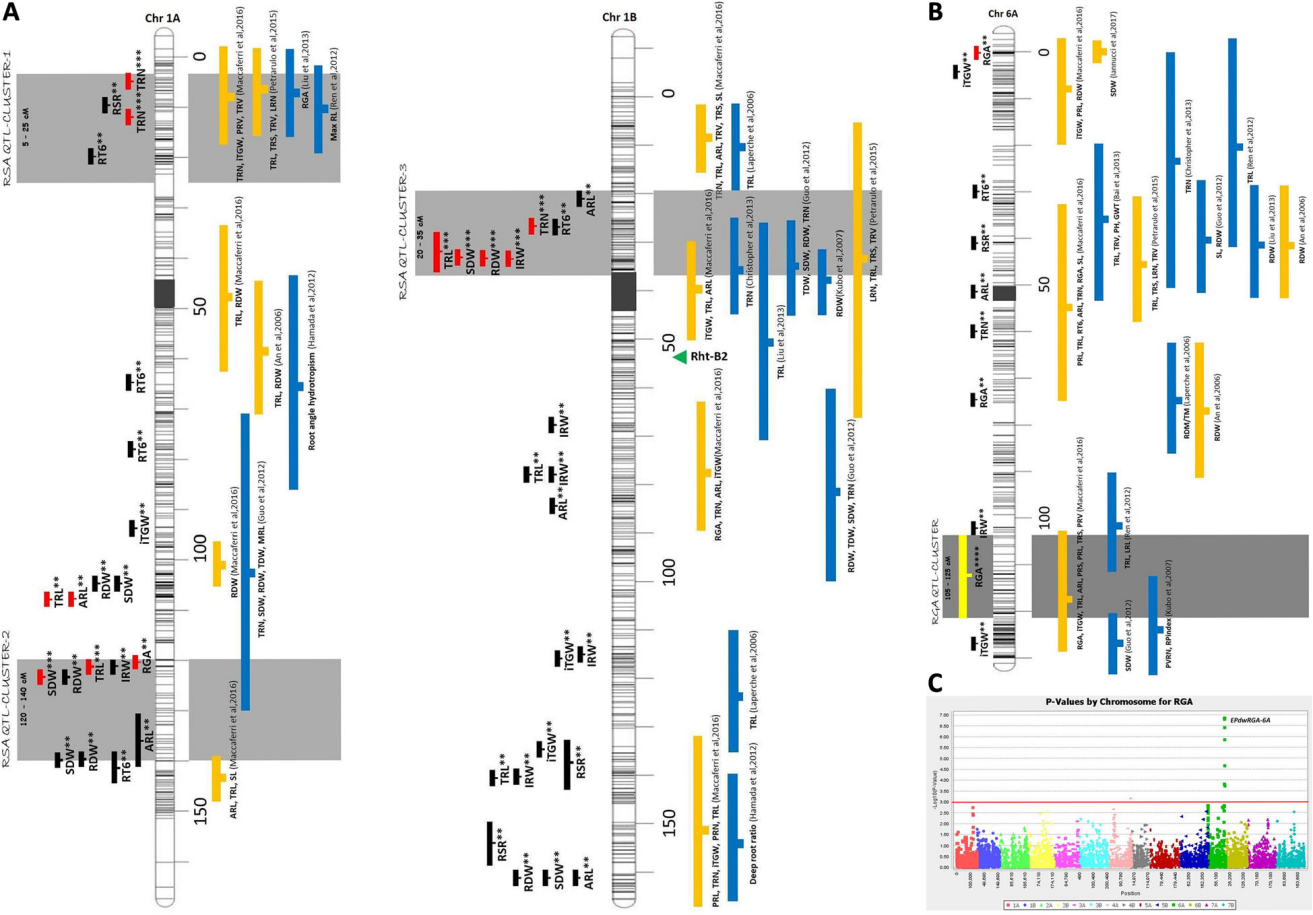
Genetic map of RSA QTLs identified in 192 Ethiopian durum wheat accessions along with previously published studies projected onto SNP-based tetraploid consensus map published in Maccaferri et al. (2015). RSA QTL identified in the present study are listed on the left side of the chromosomes with their significance level: ** = marker-wise significance of *P* ≤ 0.01 (− log_10_*P* ≥ 2); *** = marker-wise significance of *P* ≤ 0.001(− log_10_*P* ≥ 3); **** = experiment-wise significance of *P* ≤ 0.05/marker-wise significance of *P* ≤ 0.0001 (− log_10_*P* ≥ 4). RSA QTLs identified in previous studies, orange-filled bars for durum wheat and blue-filled bars for bread wheat, listed on the right side and references given in parentheses. Grey-filled bands are for RSA QTL clusters on chromosomes 1A and 1B (**a**) and a distinct root growth angle (RGA) QTL cluster identified on chromosome 6A from 105 to125 cM (**b**). Black-filled bars are for QTLs with *R*^2^ < 5%; red bars for *R*^2^ values from 5 to 10% and yellow bars for *R*^2^ > 10%. The length of bars indicates the confidence interval of each QTL or QTL cluster. Manhattan plot for the major RGA QTL identified on chromosome 6AL (**c**)

The identified QTLs were further grouped into 15 RSA QTL clusters plus one distinct RGA QTL cluster on chromosome 6AL. Clustering was based on the significance of each QTL and its effects on various traits in this study and overlapping with QTLs from previously reported studies in bread and/or durum wheat (Table 5). Based on these criteria, a total of 103 QTLs were included in 16 QTL clusters. Cluster pairs were identified on chromosomes 1A, 3B and 7A while chromosomes 1B, 2A, 2B, 3A, 4A, 4B, 5A, 5B, 6A and 6B each harbored a single QTL cluster (Fig. 4a, b and Additional file 9: Figure S5).

**Table 5:** Main RSA QTL clusters identified in 192 Ethiopian durum wheat accessions and other studies

QTL cluster	Chr	Interval (cM)	Main RSA trait			Other traits	Reference
			RSA trait	– log_10_*P*	***R***^**2**^ (%)		
*RSA QTL cluster-1*	1A	5–25	TRN	3.3	5.7	RSR, RT6	Maccaferri et al., 2016 [21]; Petrarulo et al., 2015 [36]; Ren et al., 2012 [45]
*RSA QTL cluster-2*	1A	120–140	SDW	3.3	6.3	RGA, IRW, TRL, RDW, ARL, RT6	Maccaferri et al., 2016 [21]
*RSA QTL cluster-3*	1B	20–35	TRL	3.5	6.8	ARL, TRN, RT6, IRW, RDW, SDW,	Christopher et al. 2013 [44]; Guo et al., 2012 [64] Kubo et al., 2007 [14]; Liu et al., 2013 [46]; Maccaferri et al., 2016 [21]; Petrarulo et al., 2015 [36]
*RSA QTL cluster-4*	2A	35.6	ARL	3.0	5.7	IRW, RDW, SDW, TRL	Maccaferri et al., 2016 [21]
*RSA QTL cluster-5*	2B	160–185	IRW	3.5	6.7	RGA, RDW, RT6, SDW, TRL, ARL	Guo et al., 2012; [64]Maccaferri et al., 2016 [21]
*RSA QTL cluster-6*	3A	70–100	RDW	3.2	5.8	TRN, IRW, SDW, RSR	Ren et al., 2012; [45]Maccaferri et al., 2016 [21]
*RSA QTL cluster-7*	3B	40–65	SDW	3.1	5.9	TRL, RDW, IRW, RGA	Atkinson et al., 2015 [67]; Liu et al., 2013; [46]Maccaferri et al., 2016 [21]
*RSA QTL cluster-8*	3B	120–150	iTGW	4.2	8.7	TRN, RT6, RSR	Maccaferri et al., 2016 [21]
*RSA QTL cluster-9*	4A	15–25	RDW	4.3	8.4	TRN, SDW, RT6	Maccaferri et al., 2016 [21]
*RSA QTL cluster-10*	4B	80–115	TRN	3.6	6.9	RDW, RT6, SDW, IRW, TRL	Iannucci et al., 2017; [37]Liu et al., 2013 [46]; Maccaferri et al., 2016 [21]
*RSA QTL cluster-11*	5A	0–20	IRW	4.1	8.0	TRL, RSR, RDW	Maccaferri et al., 2016 [21]; Laperche et al., 2006 [63]
*RSA QTL cluster-12*	5B	10–40	IRW	3.2	5.9	iTGW, RSR, TRN, RGA	Maccaferri et al., 2016; [21]Guo et al., 2012 [64]
^a^*RGA QTL cluster*	6A	105–125	RGA	6.9	16.1		Maccaferri et al., 2016 [21]
*RSA QTL cluster-13*	6B	75–95	IRW	3.3	6.3	ARL, iTGW, SDW, TRL	Guo et al., 2012 [64]; Maccaferri et al., 2016 [21]
*RSA QTL cluster-14*	7A	85–110	iTGW	3.4	6.7	ARL, RDW, SDW, TRL, IRW	Liu et al., 2013 [46]; Maccaferri et al., 2016 [21]
*RSA QTL cluster-15*	7A	140–150	TRN	3.1	5.7	iTGW, RT6, IRW, RGA	Guo et al., 2012; [64]Maccaferri et al., 2016 [21]

^a^A distinct RGA QTL clusters identified on chromosome 6A

See Table 6 for trait abbreviations

### QTL for seminal root length and number

**Fig. 5:**
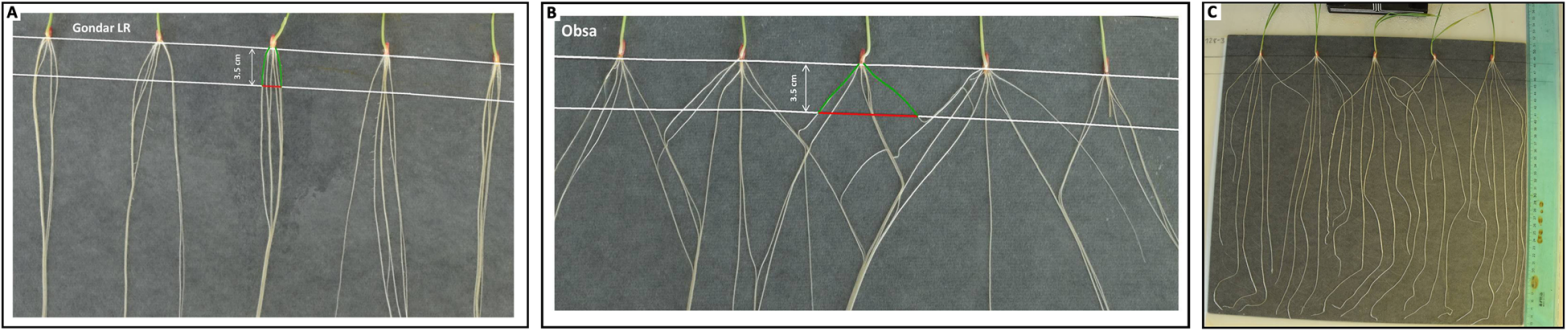
Root growth angle of seminal roots in 12-day-old seedlings of ‘Gondar’ landrace with narrow growth angle (**a**) and ‘Obsa’ cultivar with wide growth angle (**b**) measured as the linear distance (red segment) of the two most external roots (green segments) at 3.5 cm from the tip of the seed and later converted into degrees. Example of a root sample ready for image capturing for further root length and root growth angle measurement (**c**)

*EPdwTRL-1B*, *EPdwTRL-4B* and *EPdwTRL-5A* were the three nominal QTLs identified for TRL on chromosomes 1B (Fig. 4**a**), 4B and 5A, respectively. Other suggestive TRL QTLs were identified on all chromosomes except for chromosome 6A. For ARL, only one nominal QTL (*EPdwARL-2A*) was detected on chromosome 2A, while other suggestive QTLs were detected for across all chromosomes. Seven nominal QTLs were detected for TRN: three (*EPdwTRN-4B.1*, *EPdwTRN-4B.2* and *EPdwTRN-4B.3*) were mapped on chromosome 4B, two (*EPdwTRN-1A.1* and *EPdwTRN-1A.2*) on chromosome 1A (Fig. 4a) and the other two (*EPdwTRN-1B* and *EPdwTRN-7A*) on chromosomes 1B and 7A, respectively. For the presence of the sixth seminal root, three nominal QTLs (*EPdwRT6-4B.1*, *EPdwRT6-4B.2* and *EPdwRT6-4B.3*) were mapped on chromosome 4B (Table 3**).** The allelic distribution and frequency of TRN and TRL QTL-tagging SNPs with phenotypic effect (*R*^2^) > 5% are reported in Additional file 6: Table S6 and Additional file 7: Table S7, respectively.

### QTL for seminal root growth angle

The QTL with the largest effect (*R*^2^ = 0.16) on RGA (*EPdwRGA-6A*) was identified on chromosome 6A. Within the confidence interval of this QTL, six SNPs (*IWB35245*, *IWB71122*, *IWB24306*, *IWB57413*, *IWB10077* and *IWB74235*) showed significant effects for the trait (Fig. 4**c**; Additional file 4: Table S4). The confidence interval of this major RGA QTL (from 105 to 125 cM) overlapped with the confidence interval of RSA QTLs previously reported in the same region (Fig. 4b**)**. Other suggestive RGA QTLs were identified on chromosomes 1A, 2B, 3A, 3B, 4A, 5B, 6B, 7A and 7B (Additional file 4: Table S4). Notably, RGA QTLs showed no clustering with other RSA QTLs. The allelic distribution and frequency of RGA QTL-tagging SNPs with phenotypic effect > 5% is reported in Additional file 5: Table S5.

### QTL for root and shoot dry weight

Two major QTLs (*EPdwRDW-4A* and *EPdwIRW-5A*) were identified for bulk and individual root dry weight on chromosomes 4A and 5A, respectively. Two nominal QTLs were identified for RDW (*EPdwRDW-1B* and *EPdwRDW-3A*) on chromosomes 1B and 3A. As to individual root weight six nominal QTLs (*EPdwIRW-1B*, *EPdwIRW-2B*, *EPdwIRW-5B.1*, *EPdwIRW-5B.2*, *EPdwIRW-6B* and *EPdwIRW-7A*) were identified on chromosomes 1B, 2B, 5B (two QTLs), 6B and 7A, respectively. Six nominal QTLs (*EPdwSDW-1A*, *EPdwSDW-1B*, *EPdwSDW-3B*, *EPdwSDW-4A*, *EPdwSDW-4B* and *EPdwSDW-5B*) were identified for SDW. The QTLs for these three traits repeatedly clustered nearby or in single QTLs (Table 5). The allelic distribution and frequency of IRW QTL-tagging SNPs with phenotypic effect > 5% is reported in Additional file 8: Table S8.

## Discussion

In the present study, 12-day-old seedlings of 192 Ethiopian durum wheat accessions, predominantly landraces, were phenotyped in controlled conditions to identify the root system architecture (RSA) QTL through GWAS analysis. Moderate to high heritability values, ranging from 67 to 91%, were recorded for all RSA traits, confirming them as potential targets for wheat improvement.

The linkage disequilibrium analyzed from 10,789 polymorphic SNPs indicated that LD decays to the threshold value of *r*^2^ = 0.3 (the generally accepted limit to detect association with a QTL) at 2.25 cM that was in agreement with the LD decay value previously detected by Liu et al. [15]. Maccaferri et al. [40, 41] specified the LD decays at 2.20 cM for the panel comprising 183 elite durum wheat cultivars and lines from Mediterranean countries, the Southwestern USA and Mexico.

The RSA QTL-clusters included either single loci with concurrent effects on different RSA traits or tightly linked loci not resolved by recombination [42], most of which overlapped with previously identified RSA QTL clusters. QTL mapping for RSA traits of wheat based on designed bi-parental populations was recently reviewed by Soriano and Alvaro [43] compiling the results of 27 bread and three durum wheat studies for a total of 754 QTLs.

Root length and number at the seedling stage are potential candidates for marker-assisted breeding applications aimed at enhancing early rooting capacity [21]. One novel QTL for TRN, *EPdwTRN-4A*, was discovered in the present study on the short arm of chromosome 4A. The other TRN QTL identified on the short arm of chromosome 1A overlaps with the TRN QTL reported by Maccaferri et al. [21]. The confidence interval of the TRN QTL on the short arm of chromosome 1B overlapped with the confidence interval of the TRN QTL identified by Christopher et al. [44] and under the 8th root metaQTL (*Root_MQTL_8*) reported by Soriano and Alvaro [43]. Other nominal TRN QTL identified on the short arm of chromosome 4B overlapped with TRN QTL reported by Ren et al. [45]. The other two TRN QTLs detected on the long arm of chromosome 4B and short arm of chromosome 7A both overlapped with a TRN QTL reported in Maccaferri et al. [21]. Chromosome 4B showed three strong QTLs (*EPdwRT6-4B.1*, *EPdwRT6-4B.2* and *EPdwRT6-4B.3*) for the development of more than five seminal roots per plantlet.

For root length, the other important trait, d three nominal QTLs were identified for TRL and one for ARL. One novel QTL for TRL, *EPdwTRL-4B*, was mapped on the long arm of chromosome 4B. The TRL QTL identified on the short arm of chromosome 1B overlaps with TRL QTL reported by Petrarulo et al. [36] and Liu et al. [46] and the other one detected on the telomeric region of chromosome 5A overlapped with a TRL QTL reported by Maccaferri et al. [21]. The nominal ARL QTL (*EPdwARL-2A*) identified on chromosome 2A with a concurrent effect on TRL, SDW, RDW and IRW, is novel since it was not reported in any of the previous studies considered for this meta-analysis based on the tetraploid consensus map.

Among the other essential RSA traits, as to root growth angle (RGA), a pivotal trait influencing RSA and its functions, the most notable QTL (*EPdwRGA-6A*) was identified on the long arm of chromosome 6A, similarly tothat reported by Maccaferri et al. [21], *QRga.ubo-6A.2*, using 183 elite cultivars and lines representing the main breeding pools from Mediterranean countries (particularly ICARDA and Italy), the Southwestern USA and CIMMYT. Additionally, Alahmad et al. [47] recently reported sizeable and highly significant effects on RGA of the same region of chromosome 6AL. The concomitant effects of the chromosome 6AL on RGA observed in widely different germplasm pool underline the importance of further studies to better characterize the effects of the different haplotypes present at this major QTL. Notably, a novel nominal RGA QTL (*EPdwRGA-4A*) was detected on the long arm of chromosome 4A.

An additional novel major RDW QTL (*EPdwRDW-4A*) with concurrent effects on SDW, TRN and TR6 was mapped on the short arm of chromosome 4A. A novel RDW QTL (*EPdwRDW-3A*) was also identified on the long arm of chromosome 3A. *EPdwSDW-3B* and *EPdwSDW-4A* were the two newly discovered nominal SDW QTLs on the short arm of chromosome 3B and long arm of chromosome 4A, respectively. Four novel IRW QTLs (*EPdwIRW-5B.1*, *EPdwIRW-5B.2*, *EPdwIRW-6B*, *EPdwIRW-7A*) were discovered on the short arm of chromosome 5B (the first two), long arm of chromosome 6B and short arm of chromosome 7A, respectively. Iannucci et al. [37] noted the absence of a clear relationship between plant height and root development and added diverse and controversial speculations from a number of previous studies which are probably due to the different conditions and growth stages in which the root traits were evaluated. Some authors reported different genetic control between shoot and root growth [35, 48, 49] while others have reported a negative correlation [50]. Bai et al. [51] investigated a set of NILs for a number of *Rht* loci/alleles and showed clear effects on both shoot and root traits.

## Conclusions

Among the four major and 34 nominal RSA QTLs identified in the current study, 14 are novel, hence showing the suitability of Ethiopian landraces for studies aimed at the dissection of the QTL and the identification of novel haplotypes. The remaining 20 RSA QTLs concomitantly identified in this and previous studies provide valuable information on their role across diverse genepools, an important prerequisite to prioritize QTLs for marker-assisted selection aimed at enhancing crop productivity based on the use of RSA traits as proxies. A cluster of RGA QTLs was identified on the long arm of chromosome 6A with a major QTL (*EPdwRGA-6A*) with a notable phenotypic effect on RGA (*R*^2^ = 0.16). This result coupled with those reported in previous RSA studies [21, 47] highlights and reinforces *EPdwRGA-6A* as a strong candidate for further studies aimed at cloning the causative sequences and identifying the beneficial haplotypes able to positively affect yield under water- or nutrient-limited conditions.

## Methods

### Plant materials

One hundred ninety-two Ethiopian durum wheat accessions were used to assemble the GWA mapping panel. The collection included 167 landraces and 25 cultivars collected and maintained as single seed descent (SSD) progenies at the Debre Zeit Agricultural Research Center (DZARC) and Sinana Agricultural Research Center (SARC) in Ethiopia.

Landrace collections were originally collected from major wheat-producing areas of Ethiopia, including Bale, Gondar, Gojjam, Shewa, Tigray and Wollo. Twelve Ethiopian durum wheat landraces currently cultivated in the USA are included in the panel. Cultivars were released in the years between 1994 and 2010 from DZARC and SARC and have been/are being cultivated in Ethiopia. Details of accessions used for the current study are summarized in Additional file 1: Table S1.

### Root system architecture phenotyping

Seminal RSA traits were characterized using the protocol described by Canè et al. [19] and later used by Maccaferri et al. [21] with minor adjustments in the present work. Seeds were first weighed to measure thousand grain weight that was later used as a covariate in order to account for maternal effects on RSA traits due to seed size. Twenty seeds per accession were treated in 0.15% Panoctine solution and dried before pre-germinating them in Petri dishes on wet-filter-paper at 28 °C for 24 h. Then, five similar seeds with homogenous seminal root emission were positioned 7-cm apart on a wet-filter-paper sheet moistened with distilled water and placed on a vertical black rectangular (42.5 × 38.5 cm) polycarbonate plate for root obscuration.

Root traits were then measured in plantlets grown in a growth chamber for 12 days at 22 °C (day)/18 °C (night) under a 16-h photoperiod and light intensity of 400 μmol m^− 2^ s^− 1^ photosynthetically active radiation (PAR). The experiment was conducted adopting a randomized complete block design (RCBD) with three independent replications grown in the growth chamber. The experimental unit included five homogenous seedlings of each accession and hence one screening plate corresponded to one genotype. Blocking was introduced to control for possible differences in growth rate and normalization of the blocking effect (linear adjustment, whenever significant) was undertaken. Due to the high number of genotypes under evaluation and the time required for root preparation and root image acquisition, genotypes were divided into sets of 25–30 accessions that were considered as blocks. Blocks included accessions phenotyped at the same date and kept on shelves in the growth chamber that are positioned at the same distance from the floor under uniform light conditions (see Additional file 9: Figure S1).

Data for the following RSA traits were taken based on single-plantlet basis (Table 6): root growth angle (RGA) measured as the linear distance between the two most external seminal roots of each plantlet at 3.5 cm from the seed tip and then converted to degrees (Fig. 5a, b**)**; total root length (TRL); average root length (ARL); total root number (TRN); presence of six seminal roots (RT6). Total root length and root growth angle were measured on plantlet images (Fig. 5c) using GIMP (GNU Image Manipulation Program) and ImageJ [52]. Average root length was estimated as total root length divided by total root number. Bulked roots and shoots from each experiment were cut and dried in an oven for 48 h to measure root dry weight (RDW) and shoot dry weight (SDW), respectively. Individual root dry weight (IRW) was derived from the result of the bulk root dry weight divided by the total root number that could be used as a proxy to measure root thickness.

**Table 6:** Summary of acronyms used for root system architecture (RSA) traits and their measuring unit

Acronyms	Traits	Measuring Unit
**RSA traits**		
TRL	Total root length	Centimeter (cm)
ARL	Average root length	Centimeter (cm)
RGA	Root growth angle	Degree (°)
TRN	Total root number	Number (no.)
RDW	Bulk root dry weight	Milligram (mg)
SDW	Bulk shoot dry weight	Milligram (mg)
IRW	Individual root dry weight	Milligram (mg)
RSR	Root to shoot ratio	Ratio
RT6	Presence of six seminal roots per seedling	Percent (%)
iTGW	Initial thousand grain weight	Milligram (mg)

### Phenotypic data analysis

Analysis of variance (ANOVA) was conducted including replications, blocks and accessions. Block effect was controlled using the mean of each set of genotypes included in the same block and used to correct the corresponding single values, whenever significant, with a linear regression method. The weight of each individual seed was used as a covariate to correct for any possible variation caused by maternal effects. In addition, the trait was subjected to GWA analysis along with other RSA traits.

Broad sense heritability (*H*^*2*^) of RSA traits was calculated with the mean values of each experiment among the three replications according to the formula:

$$ {H}^2=\frac{\sigma^2g}{\sigma^2g+{\sigma}^2e/r} $$

Where σ^2^_g_ (genetic variance) was calculated as (MS_genotypes_ – MS_residual_)/*r*; σ^2^_e_ (the residual variance) = MS_residual_, *r* the number of replications and MS the mean square value. The coefficient of variance (CV) was calculated for all RSA traits except for the presence of the 6th root, the only trait with discrete values.

### Genotypic data and imputation

A pooled tissue sample of 25 one-week-old plantlets, from the same seed source used to phenotype RSA traits, was used for genomic DNA extraction for each accession. DNeasy 96 Plant Kit (Qiagen GmbH, Hilden, Germany) was used to extract the genomic DNA. Genotyping was done with the high-density Infinium® iSelect® Illumina 90 K wheat SNP array [53] and SNP calling and clustering were made with the GenomeStudio v2011.1 software (Illumina, San Diego, CA, USA). Calls showing residual heterozygosity were assigned as a missing value. SNP markers with < 0.05 minor allele frequencies (MAF) and markers with > 0.1 missing values per accession were excluded. After filtering, imputation of the missing data was computed using Beagle 4.0 [54]. Owing to the high level of homozygosity, imputation disregarded any phased reference populations. Twenty-five markers were considered in the imputation rolling window (twice the average number of marker present in a 5 cM interval), with an overlap of a single marker, the typical number of markers included in a 0.5 cM interval. Since imputation accuracy was not improved by using other parameters, default values were kept.

The high-density consensus map of tetraploid wheat generated by Maccaferri et al. [41] was used to identify chromosome positions of SNPs and markers with unknown positions were removed.

### Population structure and kinship analysis

For population structure analysis, a Bayesian model-based (Markov Chain Monte Carlo) clustering approach was used in STRUCTURE v.2.3 [55]. Haploview v4.2 [56] “Tagger” function (based on analysis of marker pairwise *r*^2^ values) was used to select tag-SNPs for population structure analysis with a tagger filter set at *r*^2^ = 0.5 and 1496 tag-SNPs were selected.

To infer the optimal sub-populations number, an ad hoc quantity (∆K) was calculated based on the second order rate of change of the likelihood (Evanno et al., 2005) and in this analysis approach, the ∆K shows a clear peak at the ideal number of sub-populations. To perform this, 10 sub-populations with 20 independent iterations for each sub-population were done adopting an admixture model of population structure with correlated allele frequencies and 50,000 lengths burn-in period and 100,000 Markov Chain Monte Carlo (MCMC) replications after burn-in were applied for each iteration.

Additionally, the Haploview “Tagger” function was used to select tag-SNPs for kinship matrix (K) analysis with a tagger filter set at *r*^2^ = 1 and 4842 tag-SNPs were selected, calculated in TASSEL v.5.2 [57] and incorporated in the mixed linear model (MLM) along with the population structure (Q) value for GWAS analysis.

### Linkage disequilibrium (LD) and GWAS analysis

The LD *r*^2^ values between pairwise intra-chromosomal SNPs were calculated with TASSEL v.5.2 and LD decay curve was fitted by a smoothing spline regression line at the genome level according to Hill and Weir function [58] in r environment [59]. The specific critical *r*^2^ value beyond which LD is due to true physical linkage was determined by taking the 95th percentile of *r*^2^ data of unlinked marker pairs [60]. In order to control the rate of false-positive associations, a MLM model [61] with population structure and kinship covariates was applied for the GWAS analyses. Hence, all SNP markers and the phenotypic data generated for the nine RSA traits were used to conduct the MTA analysis.

Three levels of significance were introduced according to Maccaferri et al. [21] for reporting the GWAS-QTLs: (i) experiment-wise *P* ≤ 0.05 (marker-wise *P* ≤ 0.0001, − log_10_*P* ≥ 4) for “major QTLs”; (ii) marker-wise *P* ≤ 0.001 (− log_10_*P* ≥ 3) for “nominal QTLs”; (iii) marker-wise *P* ≤ 0.01, (− log_10_*P* ≥ 2) for “suggestive QTLs”. The experiment-wise threshold was established according to the number of ‘independent SNP tests’ that was estimated in Haploview using the tagger function of *r*^2^ = 0.3 [62] and the total number (816) of tag-SNPs. Bonferroni test adjusted for multiple marker tests (*P* ≤ 0.05) was equal to – log_10_*P* = 4.21 (rounded to 4.00). Hence the experiment-wise, Bonferroni-corrected significance threshold at *P* = 0.05 matched to a marker-wise threshold of – log_10_*P* ≥ 4. Significance intervals of identified QTLs were reported as the intervals after including all SNPs associated with the trait with *P* ≤ 0.01 (marker-wise) and in LD of *r*^2^ ≥ 0.3. Confidence intervals were defined based on the GWAS-QTL peak ±2.25 cM on both map sides.

The relative positions of RSA QTLs identified in this study along with other previous studies [14, 21, 34, 36, 37, 44–46, 51, 63–67] were compared based on the projected QTL peaks and confidence intervals on the tetraploid wheat consensus map [41].

## Supplementary Information

**Additional file 1: Table S1.** Accession names and types, cultivated areas, seed sources and population structure of 192 Ethiopian durum wheat accessions.

**Additional file 2: Table S2.** Phenotypic mean values of RSA traits measured for 12-day-old seedlings in Ethiopian durum wheat accessions.

**Additional file 3: Table S3.** Inference of the true numbers of subpopulations in Ethiopian durum wheat panel.

**Additional file 4: Table S4.** List of QTLs identified for RSA traits in Ethiopian durum wheat.

**Additional file 5: Table S5.** Allelic distribution for root growth angle QTL-tagging SNPs in the Ethiopian durum wheat panel. Accessions are listed in ascending order for RGA.

**Additional file 6: Table S6.** Allelic distribution for total root number QTL-tagging SNPs in the Ethiopian durum wheat panel. Accessions are listed in ascending order for TRN.

**Additional file 7: Table S7.** Allelic distribution for total root length QTL-tagging SNPs in the Ethiopian durum wheat panel. Accessions are listed in ascending order for TRL.

**Additional file 8: Table S8.** Allelic distribution for individual root weight QTL-tagging SNPs in the Ethiopian durum wheat panel. Accessions are listed in ascending order for IRW.

**Additional file 9: Figure S1.** Introduced blocks during the root experiment in the growth chamber including accessions phenotyped at the same date and positioned shelves at the same distance from the floor under uniform light conditions. **Figure S2.** Bar chart with error bars of Ethiopian durum wheat cultivars and landraces for means of RSA traits. **Figure S3.** Box plot of the three sub-populations inferred from population structure for the mean values of RSA traits. The top and bottom of each box represent the 25th and 75th percentiles of the samples, respectively. The line in the middle of each box is the sample median. The whiskers, lines extending above and below each box, are drawn from the ends of the interquartile ranges to the farthest observations. The stars above or below the lines are outliers. **Figure S4.** Q-Q (quantile-quantile) plot results of the GWAS analysis for RSA traits using different models: General Linear Model with population structure (GLM + Q); Mixed Linear Model with population structure and kinship matrix (MLM + Q + K). **Figure S5.** Genetic map of identified RSA QTLs in Ethiopian durum wheat and previously published studies in both bread and durum wheat projected onto SNP-based tetraploid consensus map published in Maccaferri et al. (2015). RSA QTL identified in the present study are listed at the left of chromosomes with their significance level: ** = marker-wise significance of *P* ≤ 0.01 (− log_10_*P* ≥ 2); *** = marker-wise significance of *P* ≤ 0.001 (− log_10_*P* ≥ 3); and **** = experiment-wise significance of *P* ≤ 0.05/ marker-wise significance of *P* ≤ 0.0001 (− log_10_*P* ≥ 4). Black bars are for QTLs with *R*^2^ < 5%; red bars for *R*^2^ values between 5 and 10% and yellow bars for *r*^2^ > 10%. The length of bars indicates the confidence interval of each QTL and QTL cluster. The significance and colour of bars indicated is for the QTL with higher values of significance and *r*^*2*^ in the case of QTL clusters. RSA QTL from previously published studies in wheat have been projected on the consensus map and reported at the right side of chromosome bars in parentheses as orange-filled for durum wheat and blue-filled for bread wheat. The length of the bars represents the confidence interval of single QTL/cluster of QTL. Major RSA QTL-clusters of the present study are stated as grey-banded intervals.

## Data Availability

The data sets supporting the results of this article are included in this manuscript and its additional information files. The SNP markers used for the GWAS analysis can be found online at: https://bmcgenet.biomedcentral.com/articles/10.1186/s12863-020-0825-x: Additional file 2**.**

## Abbreviations

ANOVA: Analysis of variance
ARL: Average root length
DZARC: Debre Zeit Agricultural Research Center
EBI: Ethiopian biodiversity institute
GWAS: Genome-wide association study
IRW: Individual root dry weight
iTGW: Initial thousand grain weight
MAF: Minor allele frequency
MCMC: Markov chain Monte Carlo
MTA: Marker-trait association
QTL: Quantitative trait locus
RDW: Bulk root dry weight
RGA: Root growth angle
RSA: Root system architecture
RSR: Root to shoot ratio
RT6: Presence of six seminal roots per seedling
SARC: Sinana Agricultural Research Center
SDW: Bulk shoot dry weight
SNP: Single nucleotide polymorphism
TRL: Total root length
TRN: Total root number
