# Supplementary material for: Genome-wide association analysis unveils novel QTLs for seminal root system architecture traits in Ethiopian durum wheat
Source: BMC Genomics. 2021 Jan 6;22:20. doi: 10.1186/s12864-020-07320-4 (PMC7789649; doi:10.1186/s12864-020-07320-4)
Supplement: Supplementary file 9 — Additional file 9: Figure S1. Introduced blocks during the root experiment in the growth chamber including accessions phenotyped at the same date and positioned shelves at the same distance from the floor under uniform light conditions. Figure S2. Bar chart with error bars of Ethiopian durum wheat cultivars and landraces for means of RSA traits. Figure S3. Box plot of the three sub-populations inferred from population structure for the mean values of RSA traits. The top and bottom of each box represent the 25th and 75th percentiles of the samples, respectively. The line in the middle of each box is the sample median. The whiskers, lines extending above and below each box, are drawn from the ends of the interquartile ranges to the farthest observations. The stars above or below the lines are outliers. Figure S4. Q-Q (quantile-quantile) plot results of the GWAS analysis for RSA traits using different models: General Linear Model with population structure (GLM + Q); Mixed Linear Model with population structure and kinship matrix (MLM + Q + K). Figure S5. Genetic map of identified RSA QTLs in Ethiopian durum wheat and previously published studies in both bread and durum wheat projected onto SNP-based tetraploid consensus map published in Maccaferri et al. (2015). RSA QTL identified in the present study are listed at the left of chromosomes with their significance level: ** = marker-wise significance of P ≤ 0.01 (− log10P ≥ 2); *** = marker-wise significance of P ≤ 0.001 (− log10P ≥ 3); and **** = experiment-wise significance of P ≤ 0.05/ marker-wise significance of P ≤ 0.0001 (− log10P ≥ 4). Black bars are for QTLs with R2 < 5%; red bars for R2 values between 5 and 10% and yellow bars for r2 > 10%. The length of bars indicates the confidence interval of each QTL and QTL cluster. The significance and colour of bars indicated is for the QTL with higher values of significance and r2 in the case of QTL clusters. RSA QTL from previously published studies in wheat have been p [file 12864_2020_7320_MOESM9_ESM.docx]

**
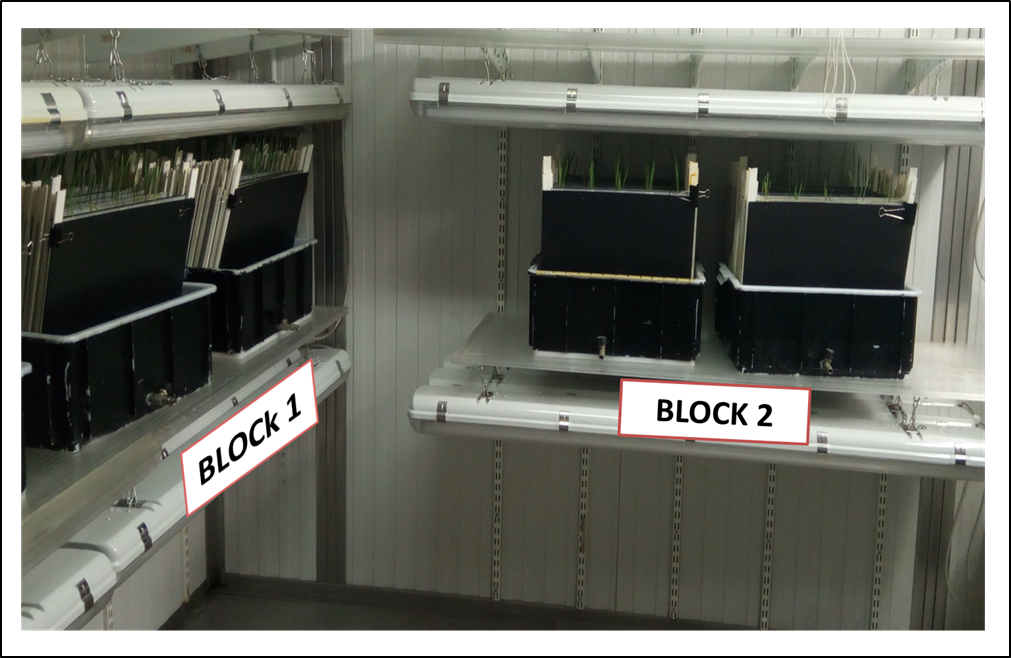
**

**Figure S1**. Introduced blocks during the root experiment in the growth chamber including accessions phenotyped at the same date and positioned at shelves at the same distance from the floor under uniform light conditions.


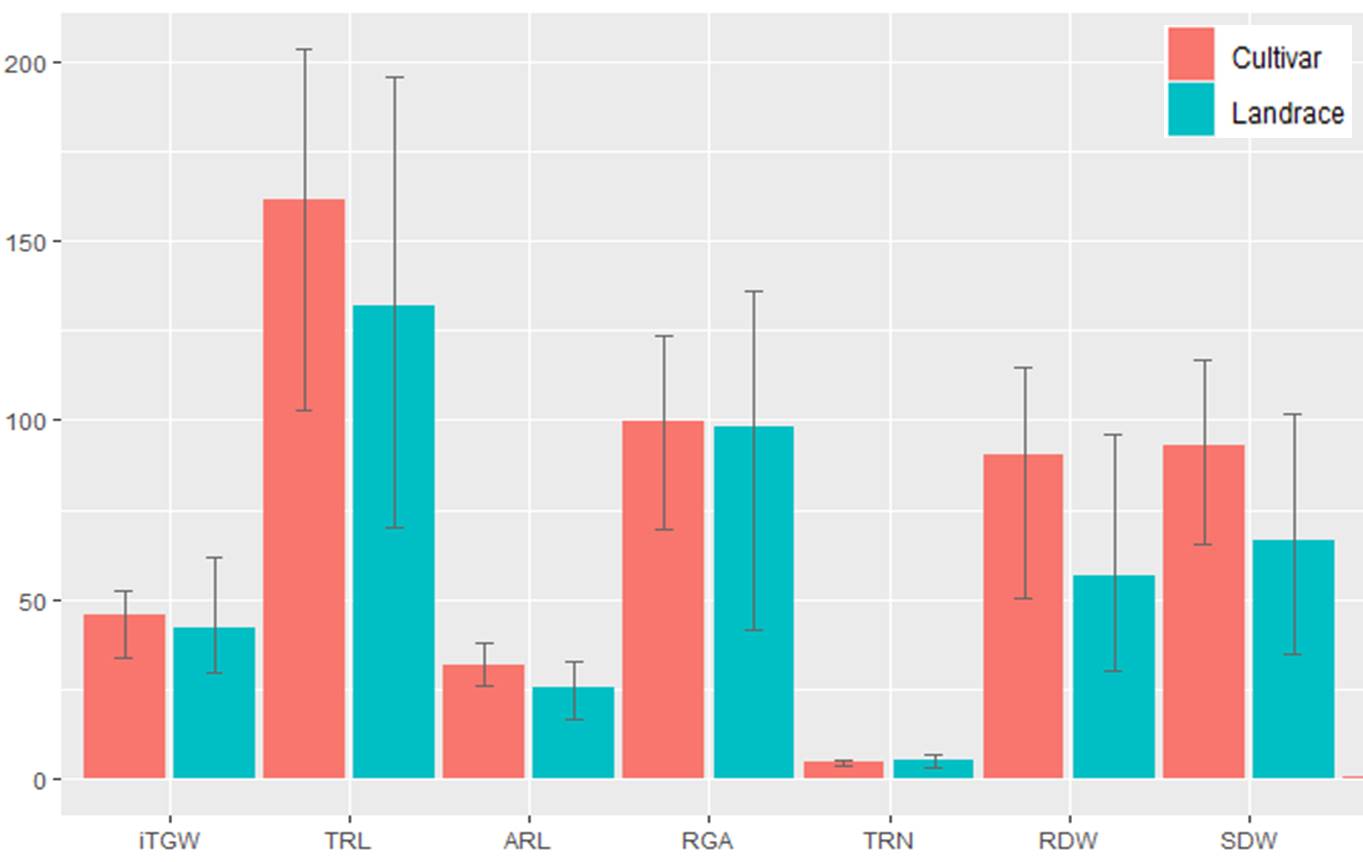


**Figure S2.** Bar chart with error bars of Ethiopian durum wheat cultivars and landraces for means of RSA traits.


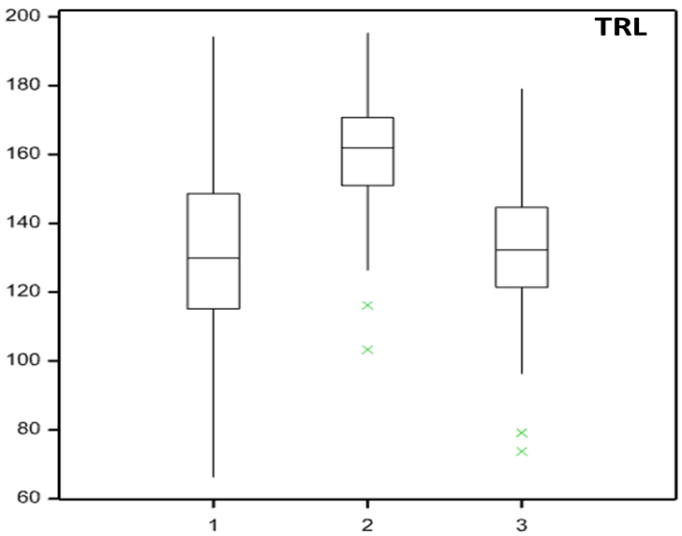

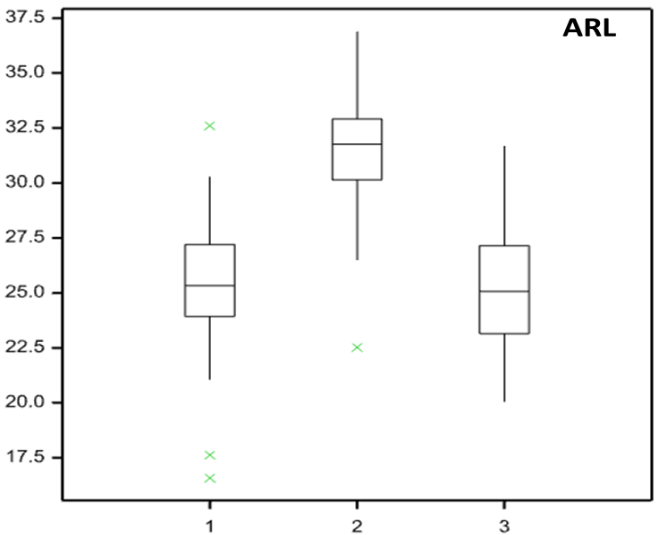

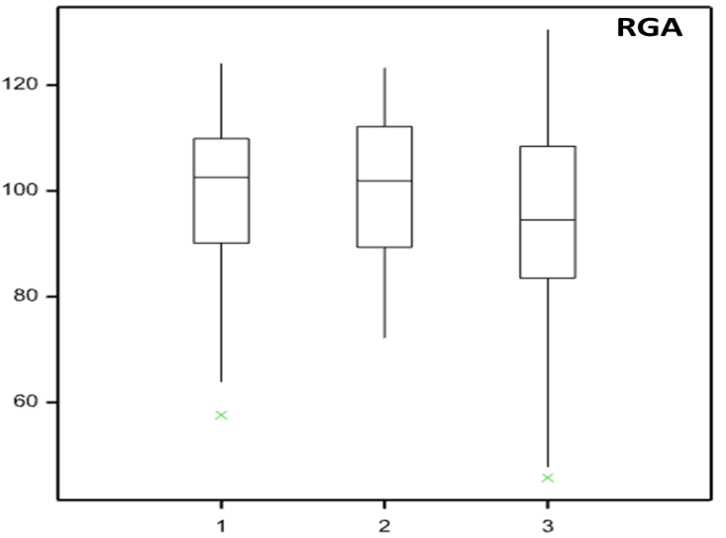

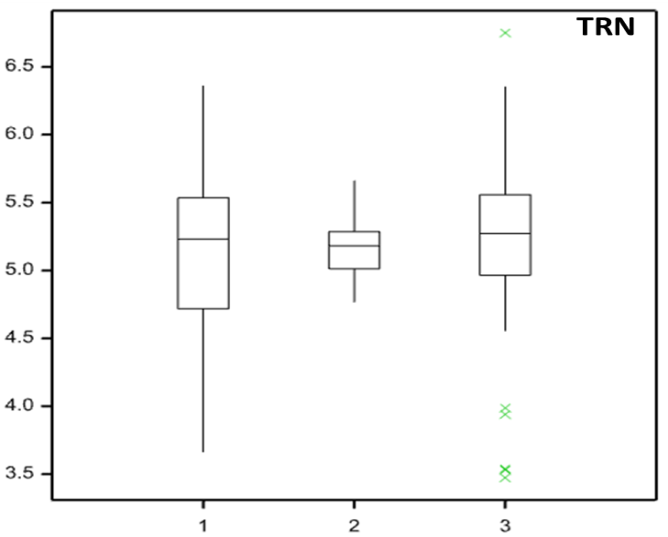

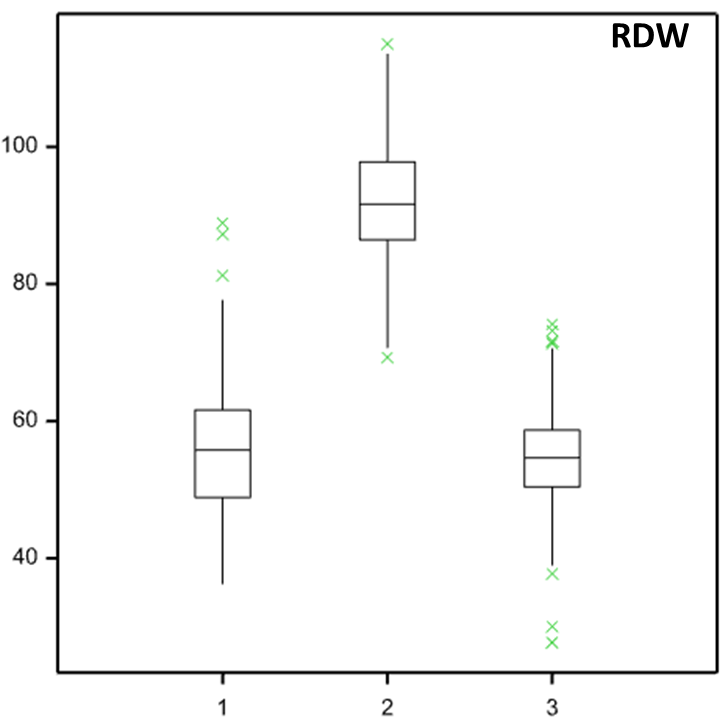

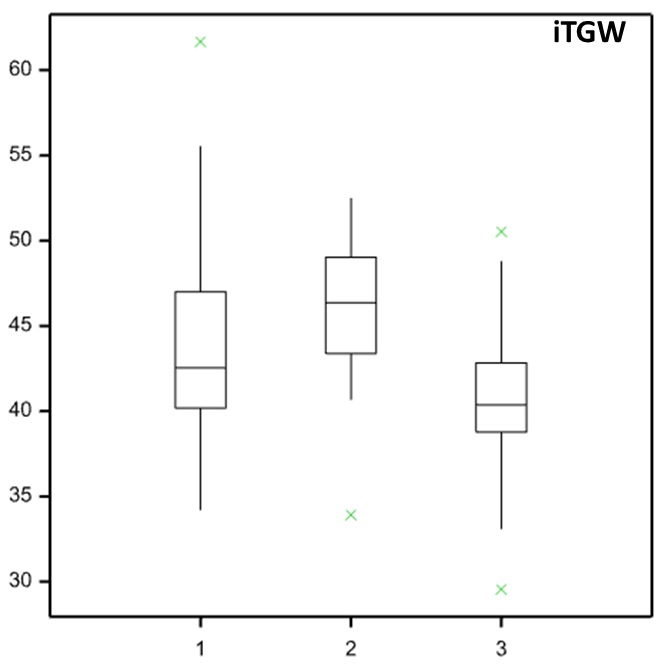


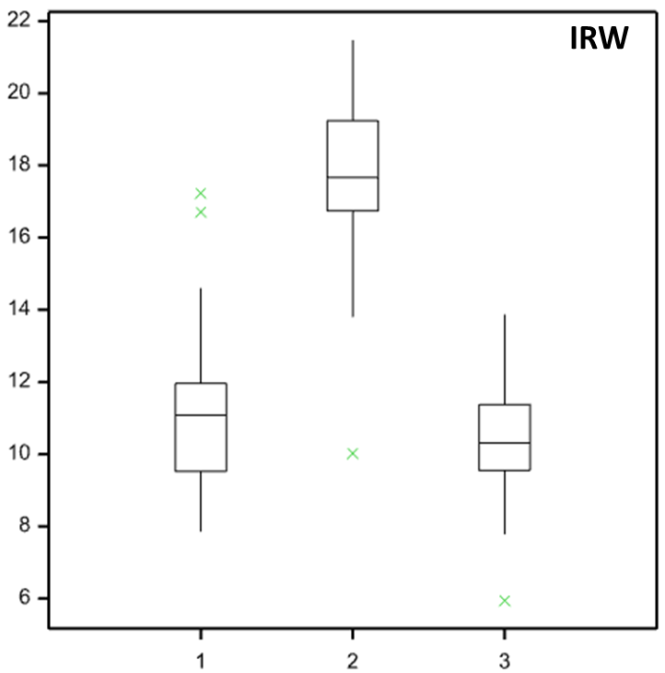

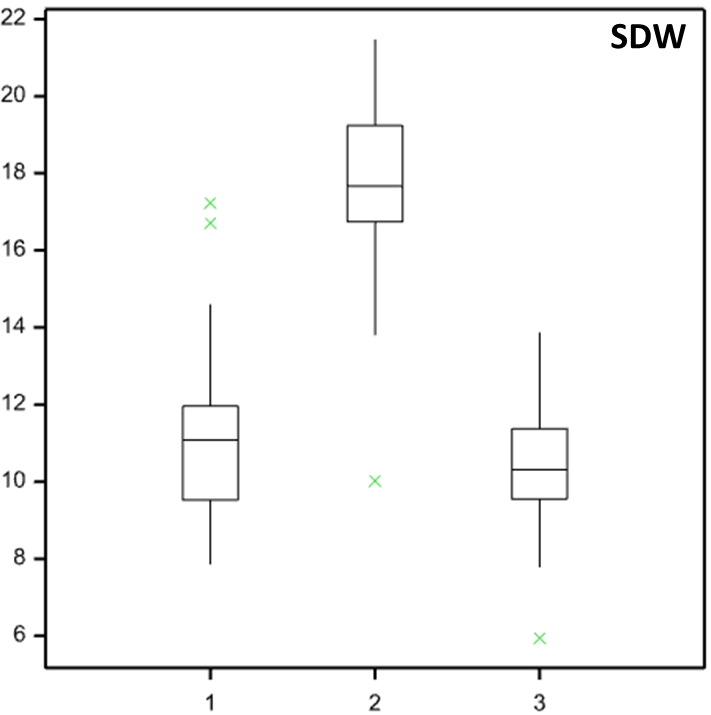

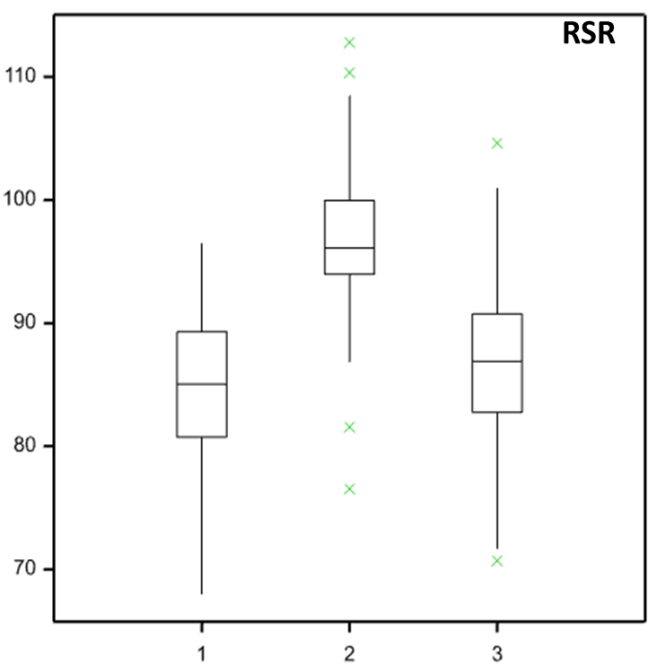

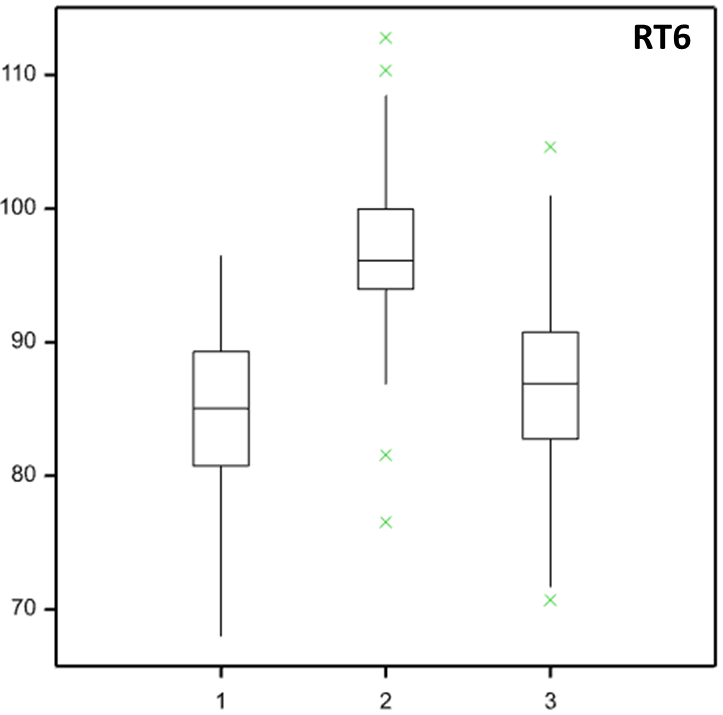


**Figure S3**. Box plot of the three sub-populations inferred from population structure for the mean values of RSA traits. The tops and bottoms of each box are the 25^th^ and 75^th^ percentiles of the samples, respectively. The line in the middle of each box is the sample median. The whiskers, lines extending above and below each box, are drawn from the ends of the interquartile ranges to the furthest observations. The starts found above or below the lines are outliers.


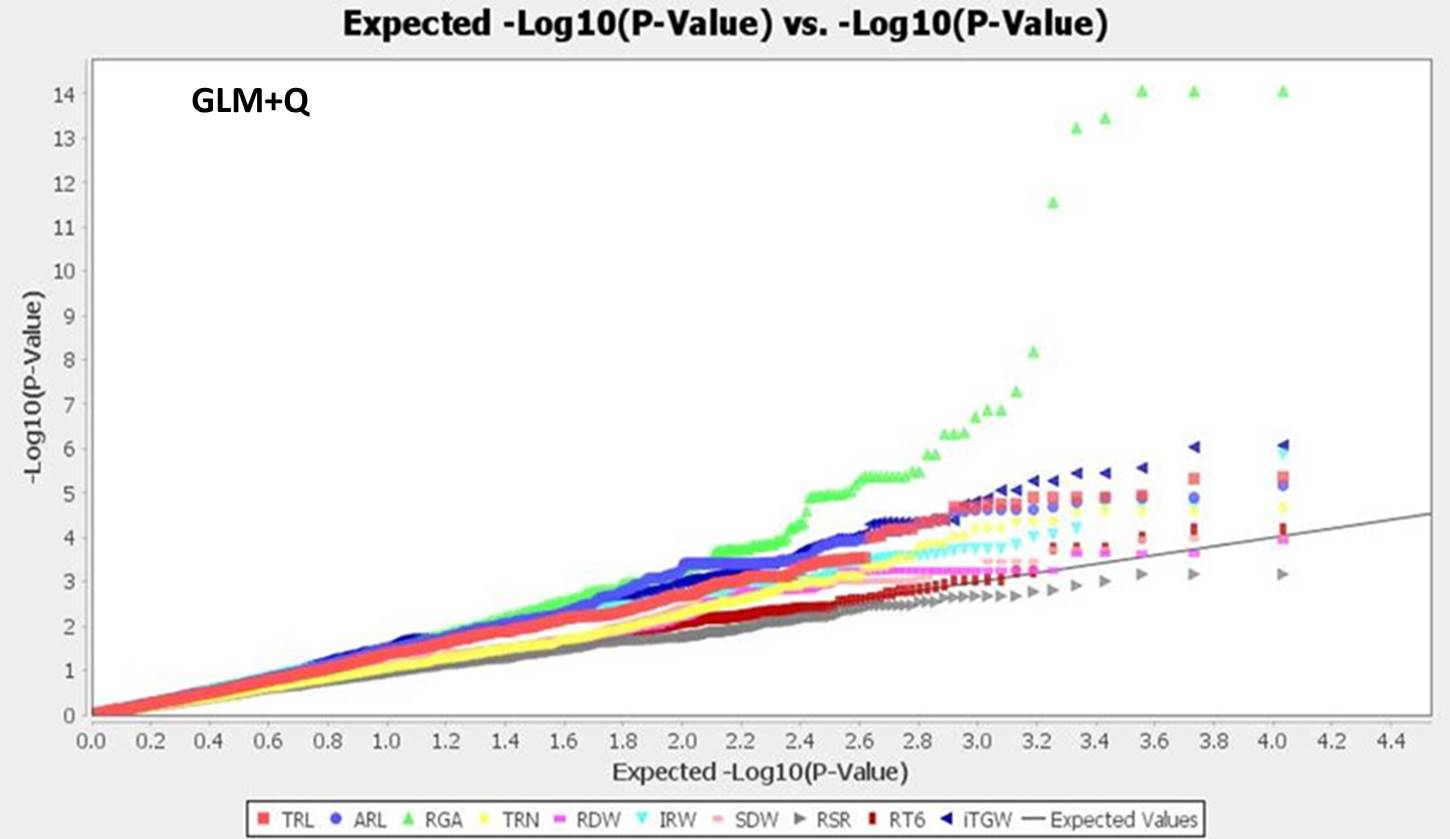


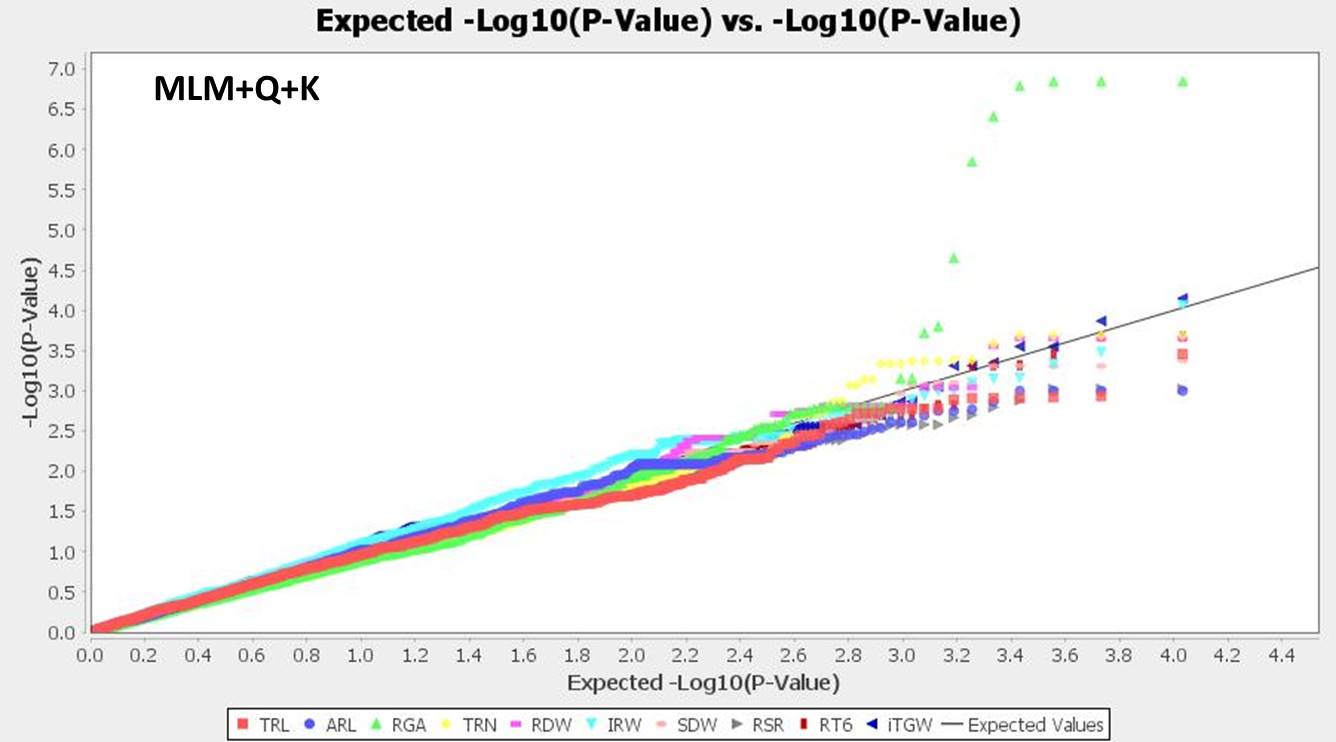


**Figure S4**. Q-Q (quantile-quantile) plot results of the GWAS analysis for RSA traits using different models: General Linear Model with population structure (GLM+Q); Mixed Linear Model with population structure and kinship matrix (MLM+Q+K).


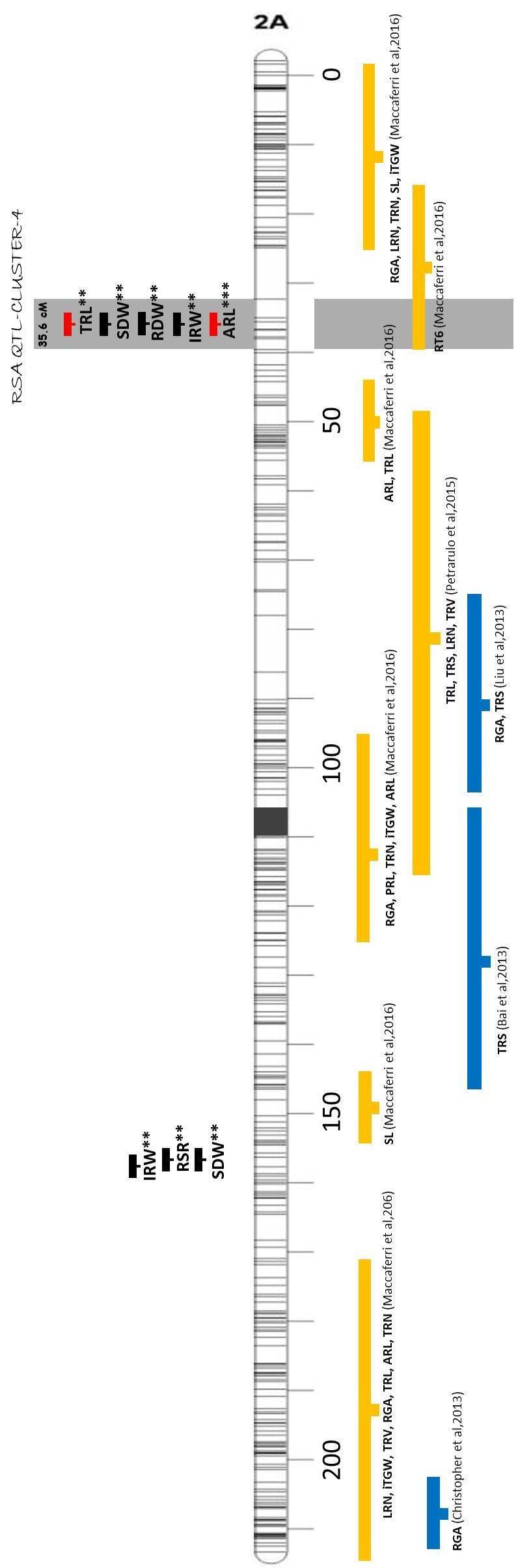

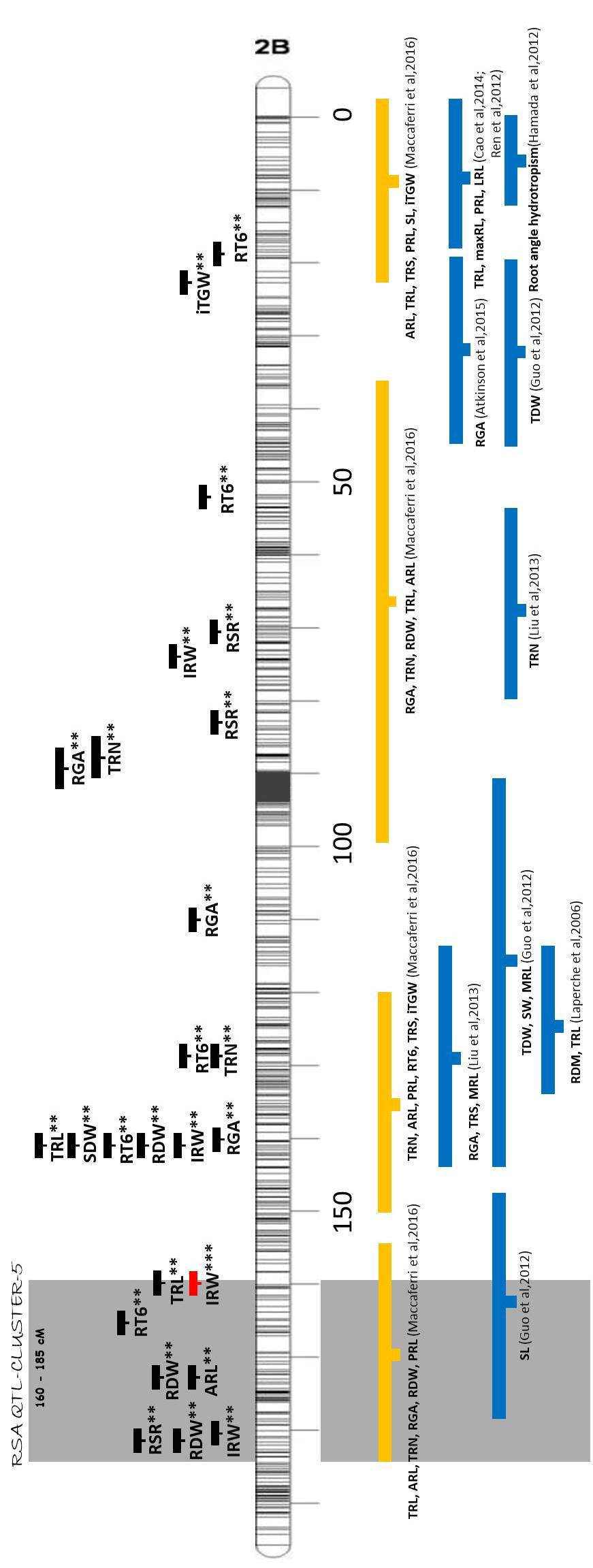


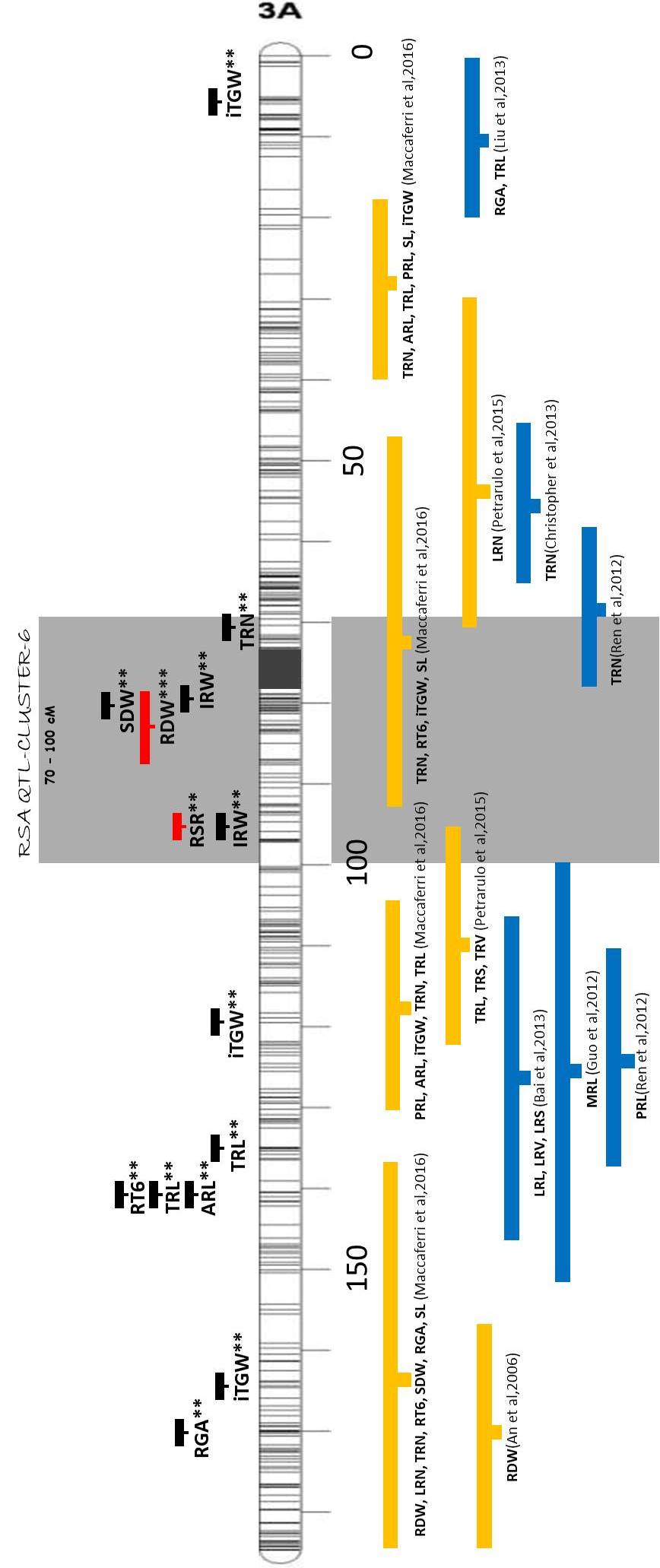

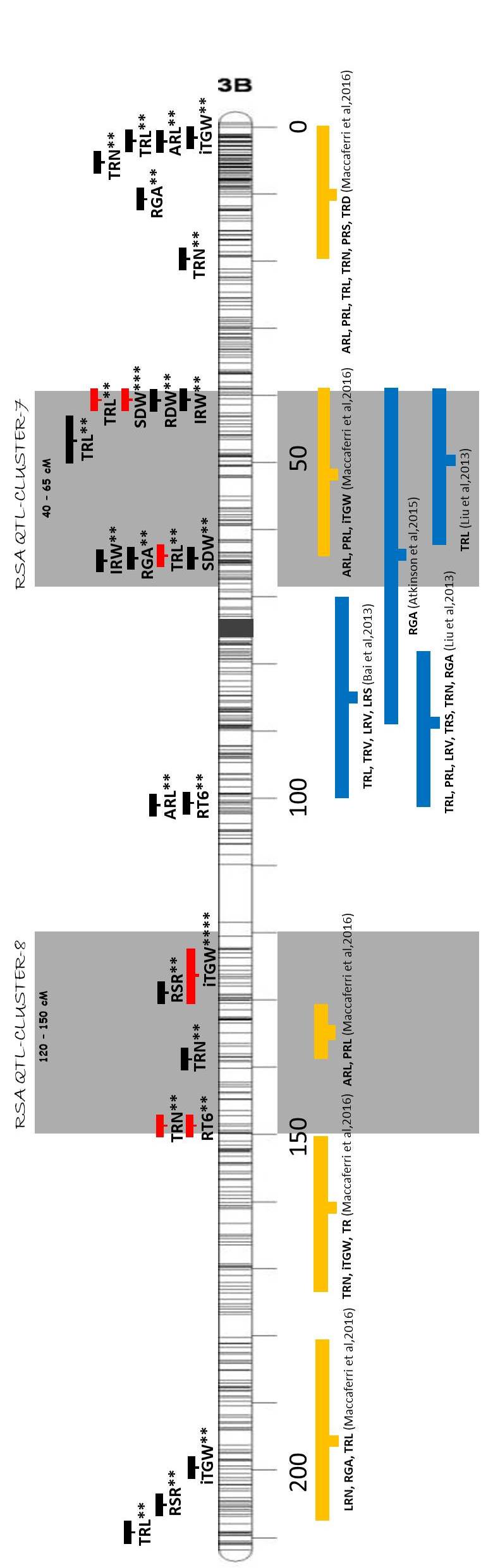


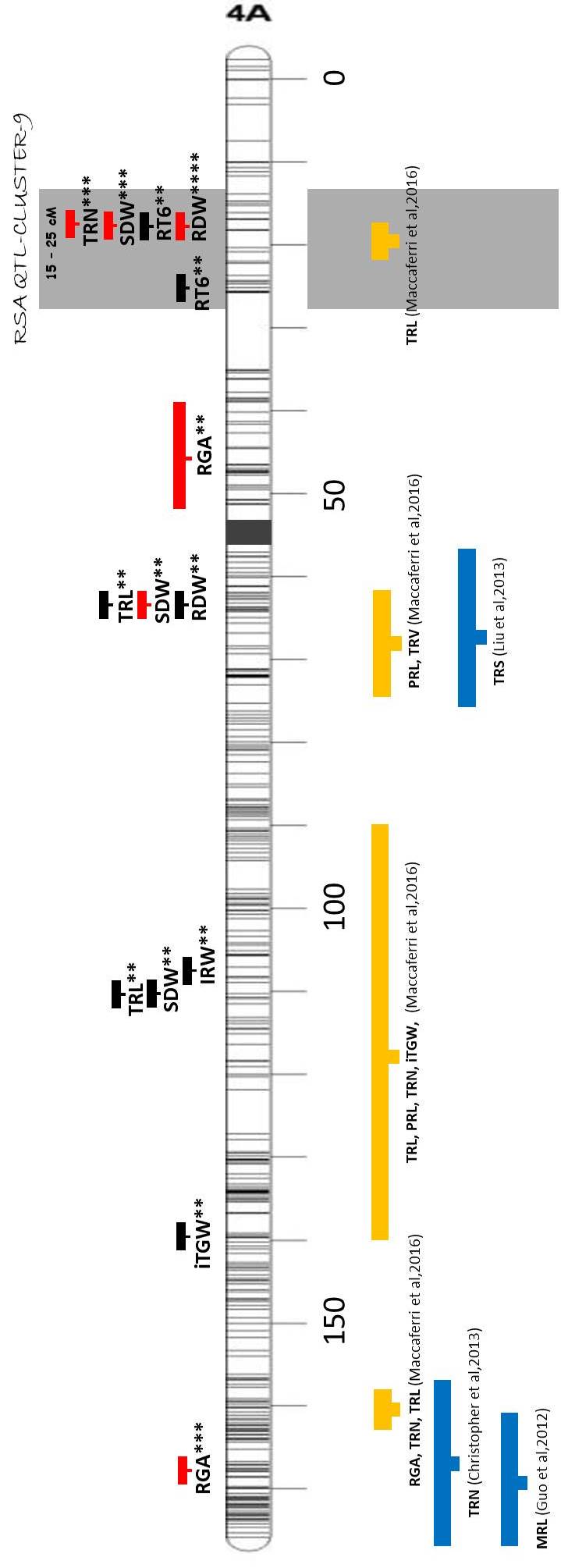

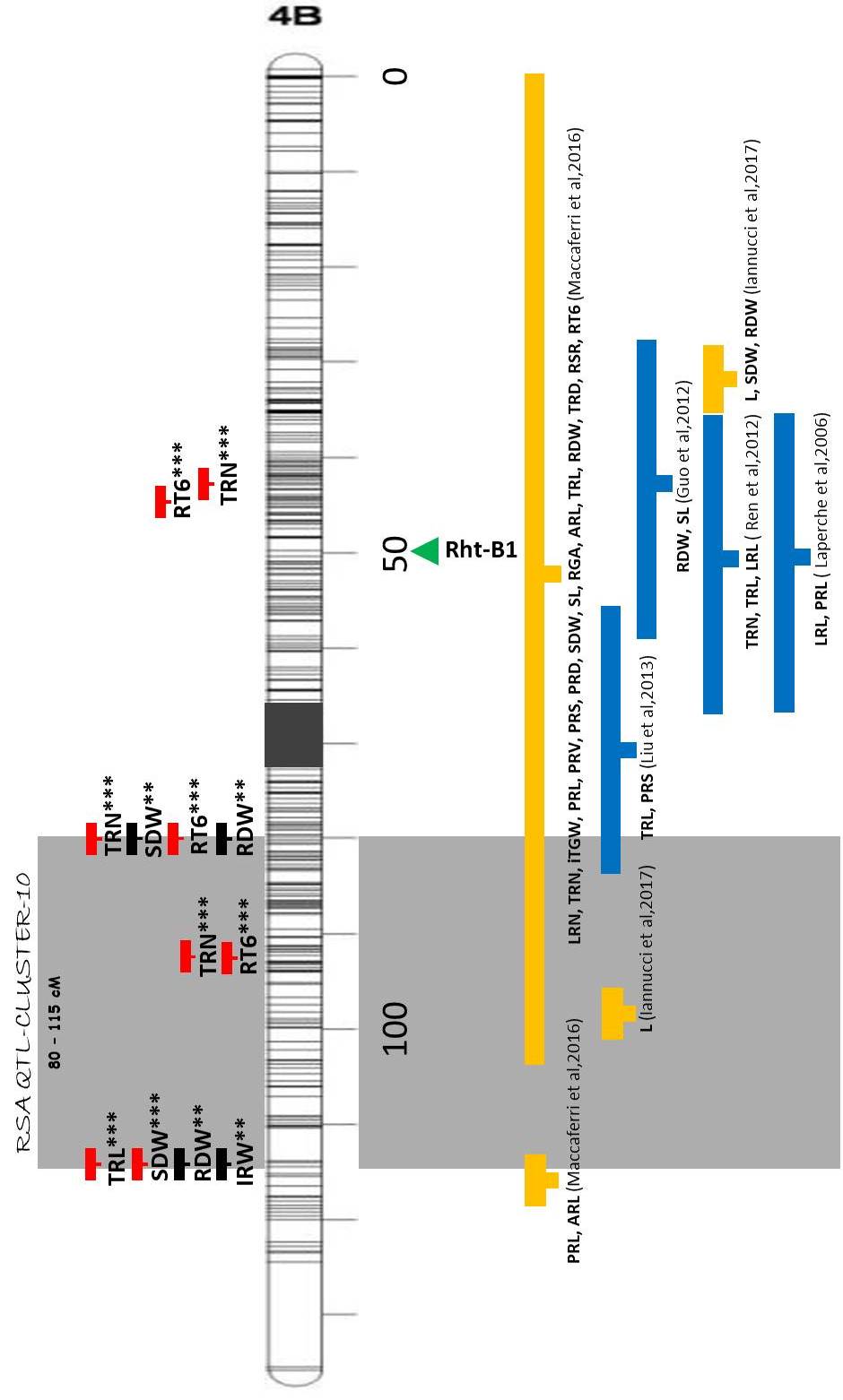


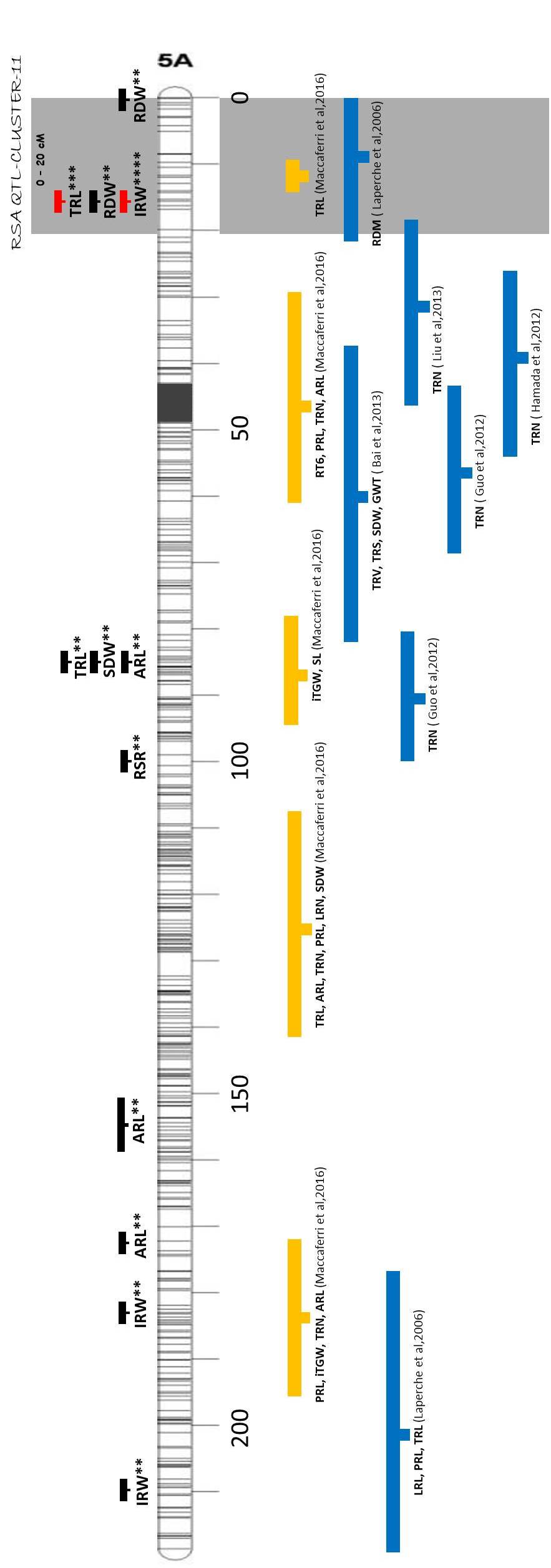

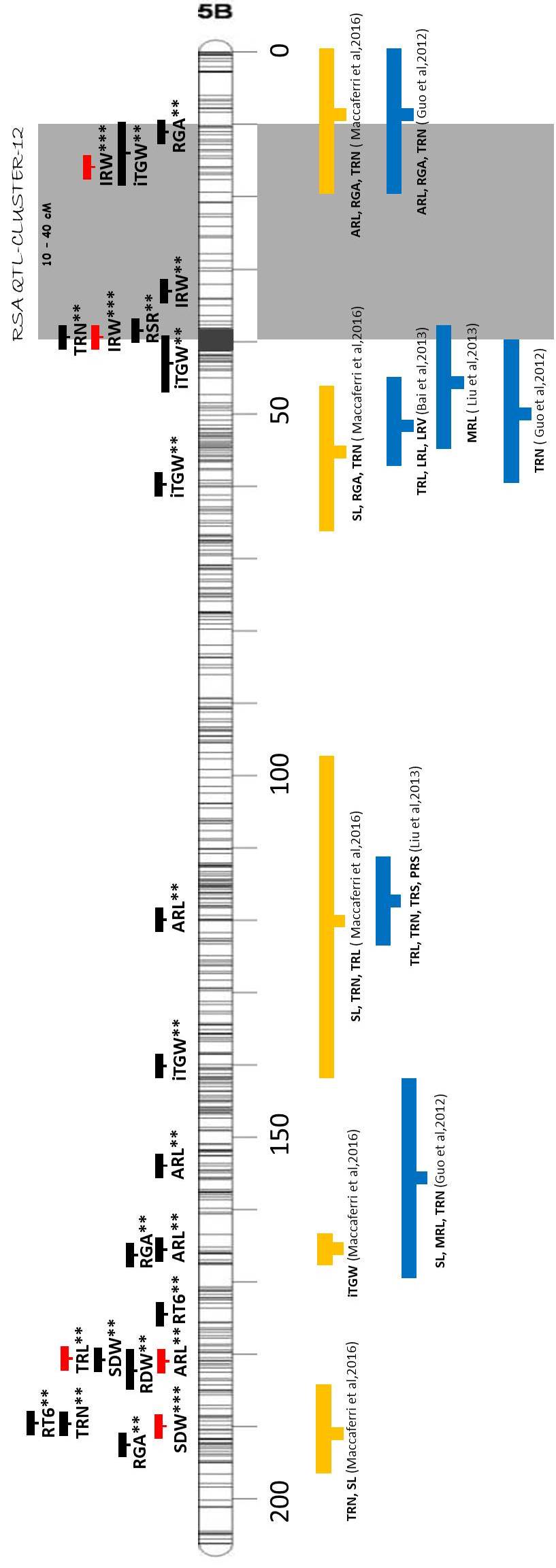


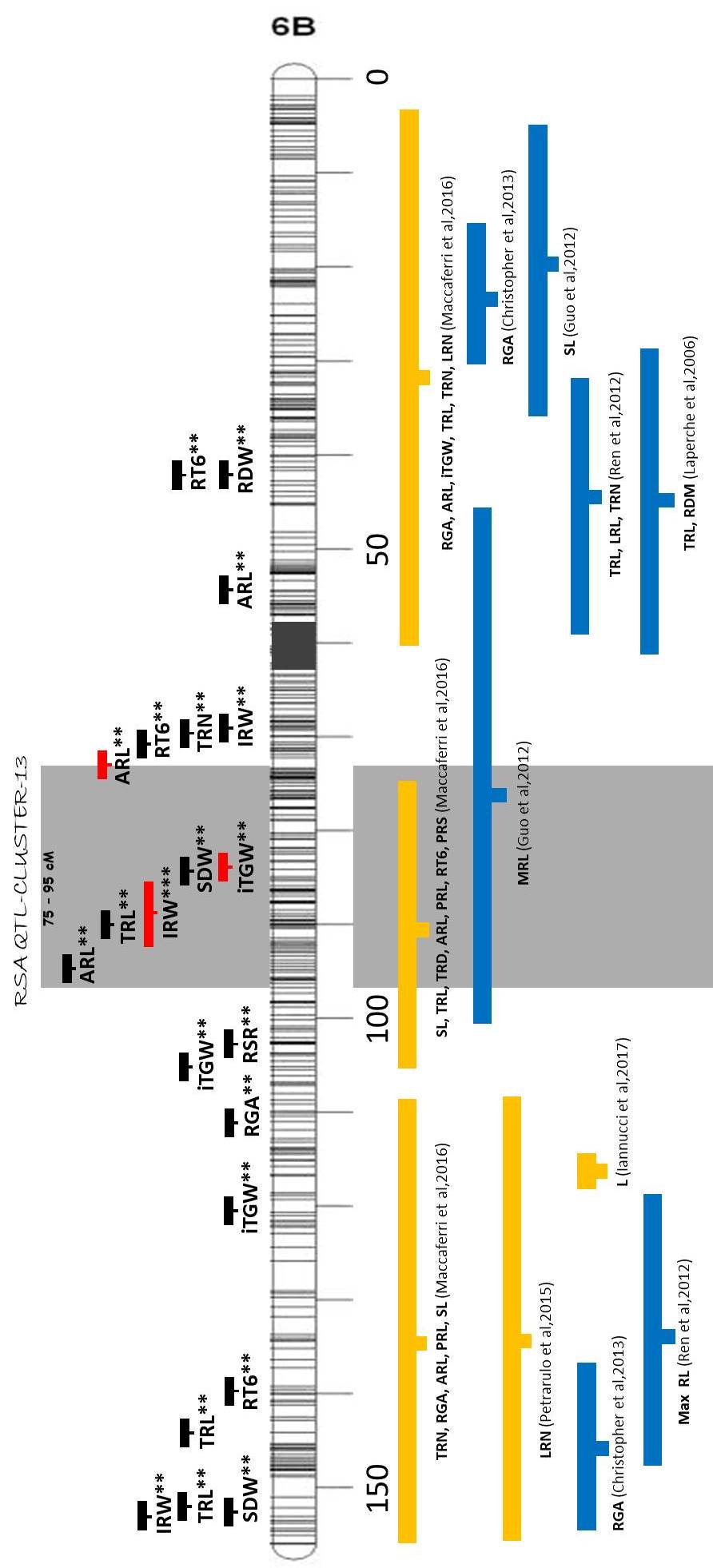

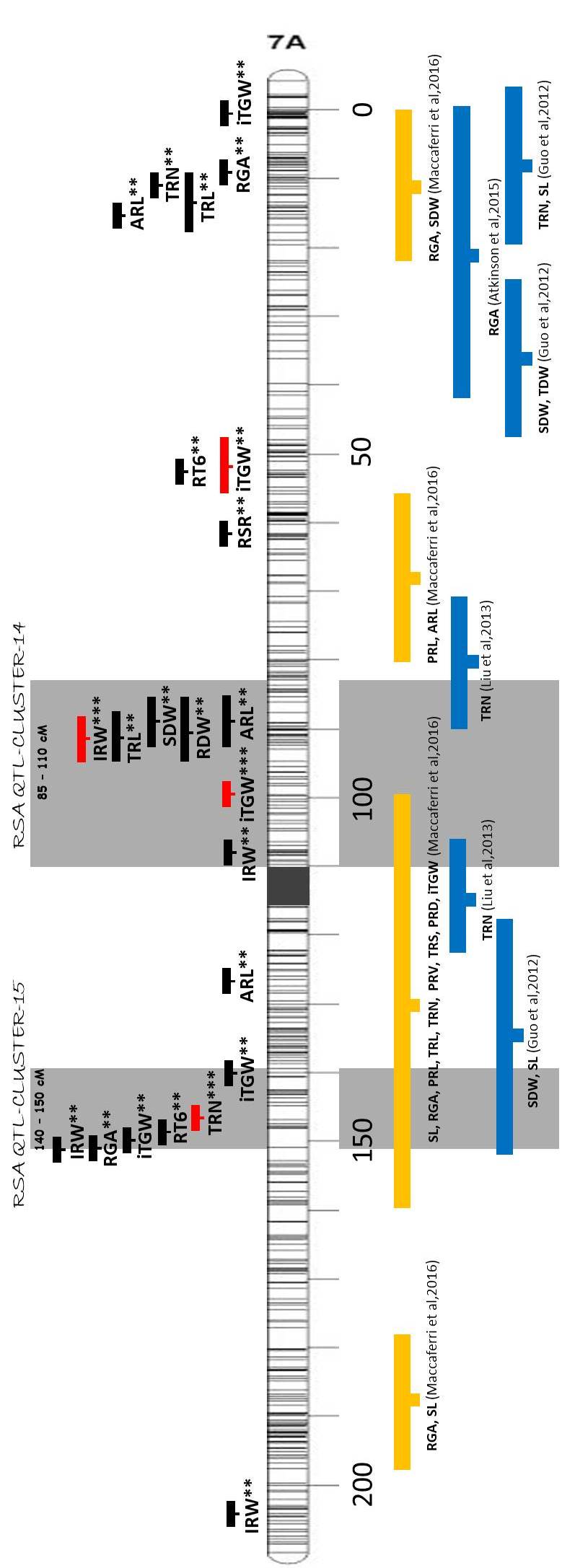


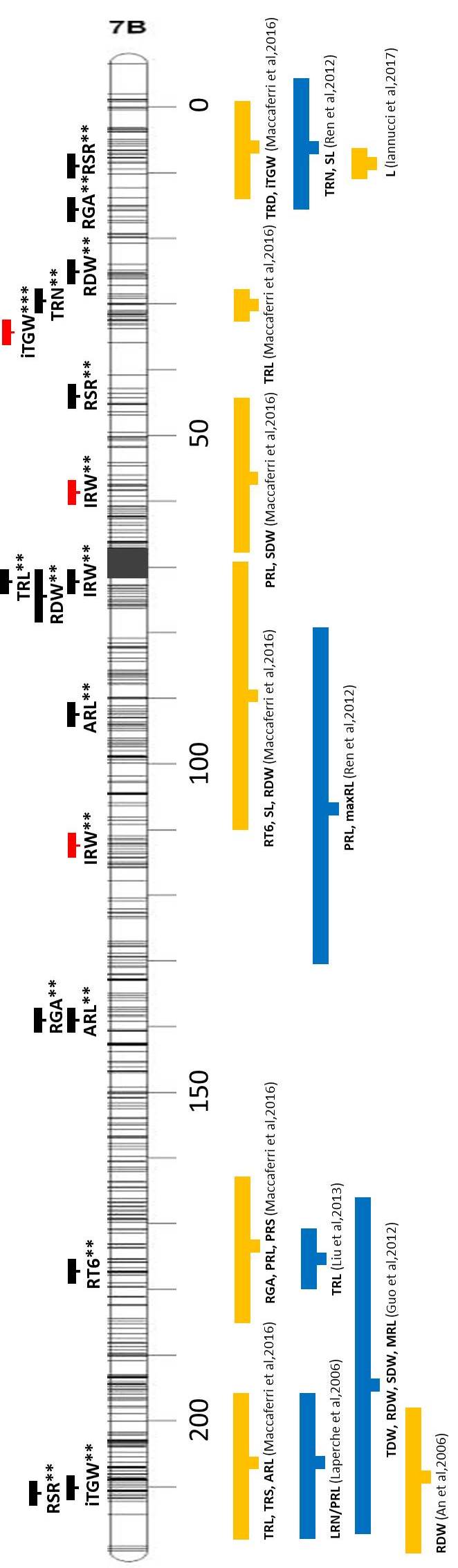


**Figure S5. .** Genetic map of identified RSA QTLs in Ethiopian durum wheat and previously published studies in both bread and durum wheat projected onto SNP-based tetraploid consensus map published in Maccaferri *et al.* (2015). RSA QTL identified in the present study are listed at the left of chromosomes with their significance level: ** = marker-wise significance of *P* ≤ 0.01 (–$\log_{10} P$≥ 2); *** = marker-wise significance of *P* ≤ 0.001(–$\log_{10} P$≥ 3); and **** = experiment-wise significance of P ≤ 0.05/ marker-wise significance of *P* ≤ 0.0001 (–$\log_{10} P$≥ 4). Black bars are for QTLs with *R*² < 5%; red bars for *R*² values between 5 and 10% and yellow bars for *r*^2^ > 10 %. The length of bars indicates the confidence interval of each QTL and QTL cluster. The significance and colour of bars indicated is for the QTL with higher values of significance and *r^2^* in the case of QTL clusters. RSA QTL from previously published studies in wheat have been projected on the consensus map and reported at the right side of chromosome bars in parentheses as orange-filled for durum wheat and blue-filled for bread wheat. The length of the bars represents the confidence interval of single QTL/cluster of QTL. Major RSA QTL-clusters of the present study are stated as grey-banded intervals.
